# Lysyl hydroxylase 2 mediated collagen post-translational modifications and functional outcomes

**DOI:** 10.1038/s41598-022-18165-0

**Published:** 2022-08-22

**Authors:** Masahiko Terajima, Yuki Taga, Tomoyuki Nakamura, Hou-Fu Guo, Yukako Kayashima, Nobuyo Maeda-Smithies, Kshitij Parag-Sharma, Jeong Seon Kim, Antonio L. Amelio, Kazunori Mizuno, Jonathan M. Kurie, Mitsuo Yamauchi

**Affiliations:** 1 Division of Oral and Craniofacial Health Sciences, Adams School of Dentistry, University of North Carolina at Chapel Hill, Chapel Hill, NC USA; 2 Nippi Research Institute of Biomatrix, Ibaraki, Japan; 3 Department of Pharmacology, Kansai Medical University, Osaka, Japan; 4 Department of Molecular and Cellular Biochemistry, University of Kentucky, Kentucky, USA; 5 Department of Pathology and Laboratory Medicine, University of North Carolina at Chapel Hill, Chapel Hill, NC USA; 6 Graduate Curriculum in Cell Biology & Physiology, Biological & Biomedical Sciences Program, UNC School of Medicine, University of North Carolina at Chapel Hill, Chapel Hill, NC USA; 7 Department of Cell Biology and Physiology, UNC School of Medicine, University of North Carolina at Chapel Hill, Chapel Hill, NC USA; 8 Biomedical Research Imaging Center, UNC School of Medicine, University of North Carolina at Chapel Hill, Chapel Hill, NC USA; 9 Lineberger Comprehensive Cancer Center, Cancer Cell Biology Program, UNC School of Medicine, University of North Carolina at Chapel Hill, Chapel Hill, NC USA; 10 Department of Thoracic/Head and Neck Medical Oncology, University of Texas MD Anderson Cancer Center, Houston, TX USA; 11 Division of Oral and Craniofacial Health Sciences, Adams School of Dentistry, University of North Carolina at Chapel Hill, 385 S Columbia Street, Koury Oral Health Sciences, Chapel Hill, NC 27599 USA

**Keywords:** Post-translational modifications, Post-translational modifications

## Abstract

Lysyl hydroxylase 2 (LH2) is a member of LH family that catalyzes the hydroxylation of lysine (Lys) residues on collagen, and this particular isozyme has been implicated in various diseases. While its function as a telopeptidyl LH is generally accepted, several fundamental questions remain unanswered: 1. Does LH2 catalyze the hydroxylation of all telopeptidyl Lys residues of collagen? 2. Is LH2 involved in the helical Lys hydroxylation? 3. What are the functional consequences when LH2 is completely absent? To answer these questions, we generated LH2-null MC3T3 cells (LH2KO), and extensively characterized the type I collagen phenotypes in comparison with controls. Cross-link analysis demonstrated that the hydroxylysine-aldehyde (Hyl^ald^)-derived cross-links were completely absent from LH2KO collagen with concomitant increases in the Lys^ald^-derived cross-links. Mass spectrometric analysis revealed that, in LH2KO type I collagen, telopeptidyl Lys hydroxylation was completely abolished at all sites while helical Lys hydroxylation was slightly diminished in a site-specific manner. Moreover, di-glycosylated Hyl was diminished at the expense of mono-glycosylated Hyl. LH2KO collagen was highly soluble and digestible, fibril diameters were diminished, and mineralization impaired when compared to controls. Together, these data underscore the critical role of LH2-catalyzed collagen modifications in collagen stability, organization and mineralization in MC3T3 cells.

## Introduction

Fibrillar type I collagen is a heterotrimeric molecule composed of two α1 and one α2 chains, and is the most abundant organic matrix component in vertebrates. The molecule consists of three structural domains: a central triple helical- (helical) and two nonhelical telopeptide domains at the amino- and carboxyl termini (N- and C-telo), and the molecules are packed into fibrils in the extracellular space to provide tissues with form and stability. To perform such functions, multiple-intra- and extracellular processing steps must occur including a series of specific lysine (Lys) post-translational modifications^1^.

Inside the cell, specific Lys residues are hydroxylated to form 5-hydroxylysine (Hyl) ^2^ that can be further modified by *O*-linked glycosylation producing galactosyl-Hyl (G-Hyl) or glucosylgalactosyl-Hyl (GG-Hyl). Lys hydroxylation is catalyzed by lysyl hydroxylases 1–3 (LH1–3) encoded by Procollagen-lysine, 2-oxyglutarate, 5-dioxygenase (*PLOD 1–3*) gene^3,4^. LH1 catalyzes Lys hydroxylation in the helical domain but the involvement of LH2 and LH3 in this function is not well defined. There are two isoforms of LH2: one includes a 63 bp-exon 13A (LH2b) and another does not (LH2a)^5^. LH2b is thought to be the key telopeptidyl LH but LH2a may also perform this function^6^. Recent studies have demonstrated that the LH activities are regulated by specific endoplasmic reticulum (ER)-resident chaperone complexes^7–10^, and that defects in LHs and these regulators result in various connective tissue disorders^10–12^. Glycosylation of Hyl is catalyzed by glycosyltransferase 25 domain containing (GLT25D) 1 and 2 to form G-Hyl, then by LH3 to produce GG-Hyl^13,14^. In the extracellular space, Lys and Hyl residues in the N- and C-telo domains of the collagen molecule can be converted to aldehyde, i.e. Lys^ald^ and Hyl^ald^, respectively, by the action of lysyl oxidases (LOXs). These aldehydes then initiate a series of condensation reactions with vicinal Lys^ald^, Lys, Hyl and histidine (His) residues to form intra and intermolecular covalent cross-links^15^. The glycosylation pattern of Hyl residues that are involved in cross-linking may control the process of cross-link maturation^16,17^.

Over 20 years ago, we proposed that LH2 may function as a telopeptidyl LH^18^. This hypothesis has been supported by several investigations including gain- and loss-of-function studies^2,19–21^. By co-expressing type I collagen α1 homotrimer and individual LH isoforms in insect cells, Takaluoma et al. showed that only LH2 could hydroxylate Lys in the α1 N-telo (9^N^, i.e. 9th residue from the N-terminus)^22^ although the extent of hydroxylation was relatively low (i.e. 25%) and the effect on the α1 C-telo Lys was not determined. Furthermore, Bank’s group identified LH2 as a telopeptidyl LH^21,23^ based mainly on analysis of Hyl^ald^-derived pyridinoline (Pyr) cross-links, however, neither the telopeptidyl Lys hydroxylation nor other cross-links was examined. Using a LH2 mutant zebrafish model, Gistelinck et al. reported that Lys in the α1 C-telo of bone type I collagen (16^C^, i.e. 16th residue from the beginning of the C-telopeptide) was not hydroxylated in the mutant^24^, though neither Lys hydroxylation of the α1 and α2 N-telo domains nor the Hyl^ald^- or Lys^ald^-derived cross-links was analyzed in this study. More recently, Gistelinck et al. reported detailed type I collagen phenotypes in bone obtained from a patient with Bruck syndrome, a rare osteogenesis imperfecta with joint contracture^25^.

Accumulating evidence indicates that LH2 plays pivotal roles in the pathogenesis of Bruck syndrome, fibrosis and cancer metastasis^21,23,26–31^. However, efforts to elucidate the function of LH2 at the cellular level in mammalian systems have been hampered since LH2 null mice die at early embryonic stage (E10.5)^32^. Thus, despite the critical importance of LH2 in these pathologies, the molecular basis is still not well understood.

Here, we generated LH2 null osteoblastic cells and extensively characterized the effects of LH2 deficiency on type I collagen molecule and its functional outcomes on collagen cross-linking, solubility, fibrillogenesis and matrix mineralization.

## Results

### Generation and validation of LH2 null (KO) cells

**Figure 1:**
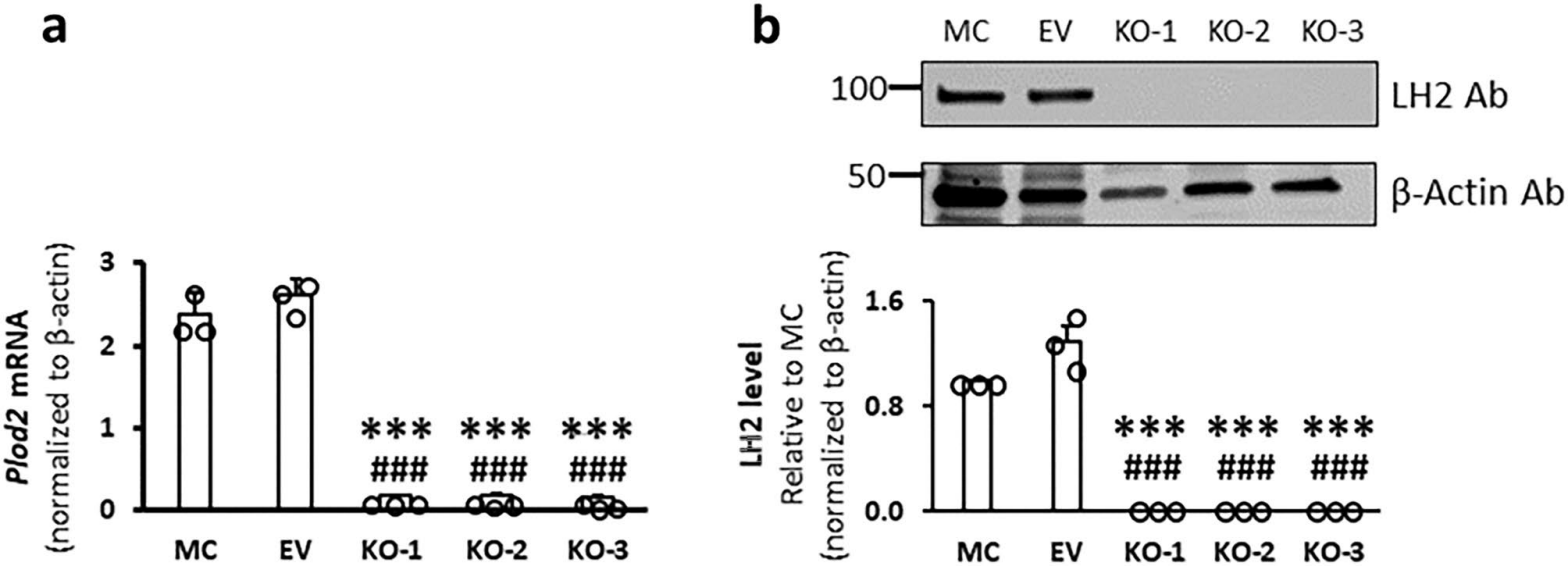
Gene expression of *Plod2* (encoding LH2) and protein levels of LH2 in MC, EV, and KO clones. (**a**) The mRNA levels relative to the internal control (*Actb*) were assessed by quantitative real-time PCR (n = 3). (**b**) The protein levels of LH2 were assessed by their immunoreactivities with the antibody (Ab) relative to that of β-actin and were then shown as the change relative to LH2 expression levels in MC as 1.0. Values represent means ± S.D. (n = 3) from three independent experiments. Statistical differences were determined by Kruskal–Wallis one way analysis of variance and means comparison with controls by Dunnett’s method. ***p < 0.001 between MC and KO; ^###^*p* < 0.001 between EV and KO, respectively. Original blot is presented in Supplementary Fig. S3. *LH* lysyl hydroxylase, *Plod* procollagen-lysine, 2-oxoglutarate 5-dioxygenase, *Actb* beta-actin, *Ab* antibody, *MC* MC3T3-E1, *EV* empty vector, *KO* knock-out.

To delete LH2, we transiently transfected MC3T3-E1 (MC) cells with plasmids expressing both Cas9 nuclease and oligonucleotides encoding sgRNAs targeting the exon 1 of the mouse *Plod2* gene. We used two different algorithms, online CRISPR RGEN Tools and Off-Spotter, to design the sgRNAs that are specific to only one gene, i.e. *Plod2*, with no other off-targets. Using *Plod2* sgRNAs, we generated three LH2 null clones (KO-1, -2 and -3) and used parental MC cells and those transfected with an empty vector (EV) as controls. To further confirm the specificity, we identified five mouse genome loci with similar sequences to *Plod2* sgRNAs using Cas-OFFinder (Supplementary Table S1). We selected KO-1 and performed Sanger sequencing of these loci (Supplementary Figs. S1, S2). No genome alteration was found, indicating that *Plod2* sgRNAs are specific. Based on the real-time PCR analysis, the LH2 mRNA levels in KO cells were 5–7% of those in controls (Fig. 1a). Western blot analysis showed that LH2 protein was not detected in any of these KO clones (Fig. 1b, Supplementary Fig. S3), thus, they were subjected to further characterization.

### Other modifying enzymes and associated proteins

**Figure 2:**
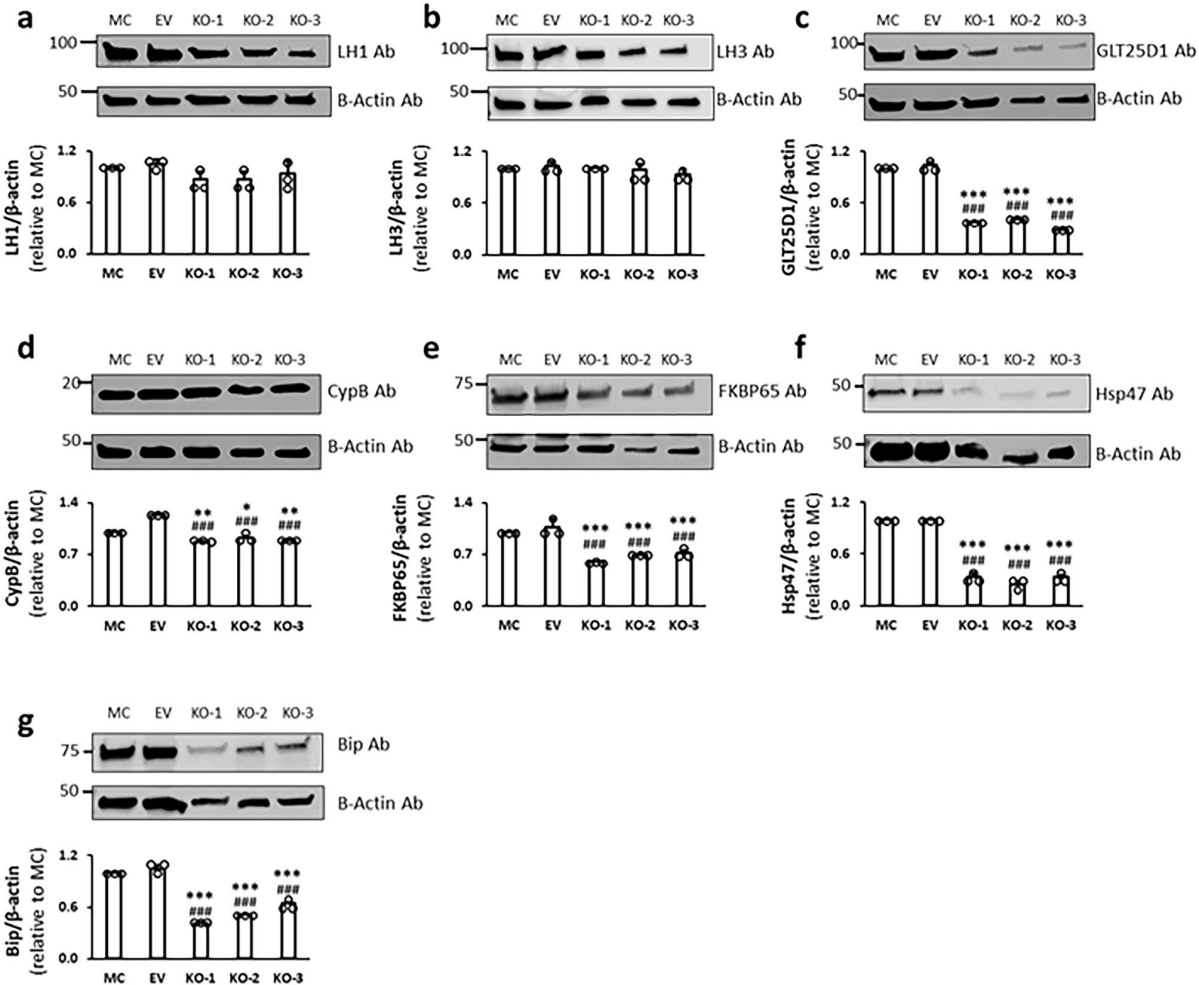
Western blot analysis for lysine modifying enzymes and chaperone complex components in cell lysates obtained from MC, EV, and KO clones. The protein levels were assessed by their immunoreactivities with the respective antibodies (Ab) relative to that of β-actin and were then shown as the change relative to LH2 expression levels in MC as 1.0. (**a**) LH1, (**b**) LH3, (**c**) GLT25D1, (**d**) CypB, (**e**) Fkbp65, (**f**) Hsp47, (**g**) Bip. Values represent mean ± S.D. (n = 3) from three independent experiments. Statistical differences were determined by the method described above (Fig. 1 legend). *p < 0.05, **p < 0.01, and ***p < 0.001 between MC and KO; ^#^*p* < 0.05, ^##^*p* < 0.01, and ^###^*p* < 0.001 between EV and KO, respectively. Original blots are presented in Supplementary Fig. S3. *LH* lysyl hydroxylase, *GLT25D1* glycosyltransferase 25 domain containing 1, *CypB* cyclophilin B, *FKBP65* FK506-binding protein 65, *Hsp47* heat shock protein 47, *Bip* immunoglobulin heavy-chain-binding protein, *Ab* antibody, *MC* MC3T3-E1, *EV* empty vector, *KO* knock-out.

We then analyzed the protein levels of LH1 and LH3 in KO clones by Western blot analysis (Fig. 2, Supplementary Fig. S3). The results showed that both LH1 and LH3 were comparable to controls (*p* > 0.05) though the former tended to be slightly lower in KO clones (Fig. 2). The collagen galactosyl transferase, GLT25D1, was significantly lower in the KO clones when compared to controls (Fig. 2). The reason for this is unclear, but the reduced level of GLT25D1 in KO could be partially compensated by unknown mechanisms since the total levels of G- + GG-Hyl in KO collagen were only slightly lower (< 10%) than those of controls at all glycosylation sites analyzed (see below). The LH2-specific chaperone, FK506-binding protein 65 (FKBP65)^10^, and an additional potential binding partner, cyclophilin B (CypB)^33^, showed slightly but significantly lower (~ 70% of controls) or similar level (~ 90%), respectively, in KO clones when compared to controls (Fig. 2). Other LH2-associated proteins, heat shock protein 47 (Hsp47) and immunoglobulin heavy-chain-binding protein (Bip)^34^, were also significantly lower in KO than controls (Fig. 2).

All these measurements were conducted using cells cultured with the standard medium, thus, further analyses using those with the differentiation and mineralization media (see below) are necessary to obtain more comprehensive information.

### Collagen type

We first examined collagen types by mass spectrometric analysis^35^. The data revealed that type I collagen is by far the predominant collagen type with a small amount of type III in all of the culture samples, which is consistent with our previous report^36^. The percentages of type I calculated by I/(I + III) × 100 were all > 96% and the difference between MC and KOs was within ~ 2% range (Table 1) demonstrating that LH2 deficiency does not alter collagen types.

**Table 1:** Collagen type analysis by mass spectrometry of cell/matrix layer from controls (MC and EV), and KO clones.

	MC	EV	KO-1	KO-2	KO-3
Type I collagen (%)	98.3	98.6	97.9^##^	96.0***^,###^	97.4***^,###^
(S.D.)	(0.04)	(0.13)	(0.10)	(0.28)	(0.08)

Percentages of type I collagen calculated by type I collagen/(type I + type III collagen).

Values represent mean the percentages of type I collagen ± S.D. (n = 3) of triplicate analysis of the hydrolysates. Statistical differences were determined by Kruskal–Wallis one-way analysis of variance and means comparison with the controls by Dunnett’s method.

*MC* MC3T3-E1, *EV* empty vector, *KO* knock-out.

***p < 0.001 between MC and KO; ^##^*p* < 0.01 and^###^*p* < 0.001 between EV and KO, respectively.

### Lys hydroxylation determined by high performance liquid chromatography (HPLC)

In KO clones, levels of Lys hydroxylation in collagen were slightly but significantly decreased compared with those from MC and EV (Table 2). We then analyzed Lys modifications at specific molecular loci in type I collagen (see below).

**Table 2:** Hydroxylation of Lys in type I collagen from controls (MC and EV), and KO clones.

	MC	EV	KO-1	KO-2	KO-3
Hyl	14.1	14.5	12.5***^,###^	13.2^###^	11.4***^,###^
(S.D.)	(0.21)	(0.34)	(0.14)	(0.12)	(0.58)

Values represent mean Hyl residues/mole of collagen ± S.D. (n = 3) of triplicate analysis of the hydrolysates. Statistical differences were determined by Kruskal–Wallis one-way analysis of variance and means comparison with the controls by Dunnett’s method.

*Lys* lysine, *Hyl* hydroxylysine, *MC* MC3T3-E1, *EV* empty vector, *KO* knock-out.

***p < 0.001 between MC and KO; ^###^*p* < 0.001 between EV and KO, respectively.

### Lys modifications at specific molecular loci in type I collagen

Relative abundance of unmodified Lys and its modified forms (Hyl, G-Hyl and GG-Hyl) at specific molecular sites of type I collagen are summarized in Table 3. Based on these values, an extent of Lys hydroxylation (%) was calculated as [Hyl/(Lys + Hyl) × 100] where Hyl is a sum of non-glycosylated, G- and GG-Hyl.

**Table 3:** Summary of site-specific modification analysis by mass spectrometry of non-cross-linked, hydroxylated and glycosylated residues in type I collagen from controls (MC and EV) and KO clones.

		Site occupancy (%)				
		MC	EV	KO-1	KO-2	KO-3
α1(I) K87	Lys	1.9 ± 0.1	1.7 ± 0.1	5.6 ± 0.1***^,###^	5.5 ± 0.2***^,###^	3.8 ± 0.0***^,###^
	Hyl	3.9 ± 0.2	3.2 ± 0.1	12.3 ± 1.2***^,###^	11.9 ± 0.2***^,###^	5.1 ± 0.1***^,###^
	G-Hyl	7.7 ± 0.2	7.8 ± 0.1	10.5 ± 0.3***^,###^	10.4 ± 0.3***^,###^	7.3 ± 0.7
	GG-Hyl	86.4 ± 0.1	87.2 ± 0.1	71.6 ± 0.5***^,###^	72.3 ± 0.6***^,###^	83.8 ± 0.6***^,###^
α1(I) K99	Lys	69.4 ± 0.4	70.5 ± 0.3	70.4 ± 0.1***^,###^	70.9 ± 0.3***^,###^	67.3 ± 0.1***^,###^
	Hyl	20.0 ± 0.4	19.7 ± 0.3	22.1 ± 0.1***^,###^	21.4 ± 0.4***^,###^	25.0 ± 0.1***^,###^
	G-Hyl	6.9 ± 0.1	6.4 ± 0.1	5.5 ± 0.1***^,###^	5.6 ± 0.1***^,###^	4.9 ± 0.1***^,###^
	GG-Hyl	3.7 ± 0.1	3.4 ± 0.0	2.0 ± 0.0*	2.1 ± 0.0***	2.8 ± 0.0***^,###^
α1(I) K174	Lys	53.3 ± 0.9	52.8 ± 0.3	64.3 ± 0.3***^,###^	64.6 ± 0.3***^,###^	57.7 ± 0.2***^,###^
	Hyl	41.2 ± 0.4	42.3 ± 0.1	33.1 ± 0.2***^,###^	32.6 ± 0.2***^,###^	38.4 ± 0.2***^,###^
	G-Hyl	3.1 ± 0.2	2.8 ± 0.2	1.9 ± 0.1***^,###^	2.0 ± 0.0***^,##^	2.5 ± 0.0*
	GG-Hyl	2.4 ± 0.3	2.1 ± 0.1	0.7 ± 0.1***^,###^	0.8 ± 0.1***^,###^	1.3 ± 0.0***^,##^
α1(I) K219	Lys	85.4 ± 0.2	87.0 ± 0.2	83.6 ± 0.3***^,###^	83.3 ± 0.3***^,###^	81.3 ± 0.2***^,###^
	Hyl	14.6 ± 0.2	13.0 ± 0.2	16.4 ± 0.3***^,###^	16.7 ± 0.3***^,###^	18.7 ± 0.2***^,###^
α1(I) K564	Lys	66.6 ± 1.2	67.2 ± 0.3	73.6 ± 0.5***^,###^	72.5 ± 0.1***^,###^	67.5 ± 0.1
	Hyl	24.1 ± 0.8	24.7 ± 0.2	22.3 ± 0.4^###^	23.1 ± 0.0^##^	27.3 ± 0.2***^,###^
	G-Hyl	4.3 ± 0.3	4.0 ± 0.2	2.6 ± 0.1***^,###^	2.8 ± 0.1***^,###^	3.0 ± 0.1***^,##^
	GG-Hyl	4.9 ± 0.2	4.2 ± 0.2	1.5 ± 0.0***^,###^	1.6 ± 0.2***^,###^	2.2 ± 0.0***^,###^
α2(I) K87	Lys	8.4 ± 0.1	7.2 ± 0.2	7.2 ± 0.1***	7.2 ± 0.1***	4.1 ± 0.0***^,###^
	Hyl	91.6 ± 0.1	92.8 ± 0.2	92.8 ± 0.1***	92.8 ± 0.1***	95.9 ± 0.0***^,###^
α2(I) K174	Lys	36.0 ± 1.4	35.6 ± 1.3	48.7 ± 0.3***^,###^	49.4 ± 0.4***^,###^	39.8 ± 0.6**^,##^
	Hyl	6.2 ± 0.4	6.0 ± 0.4	8.0 ± 0.3***^,###^	8.1 ± 0.4***^,###^	4.6 ± 0.0*^,##^
	G-Hyl	37.8 ± 0.3	38.4 ± 0.5	33.1 ± 0.7***^,###^	31.9 ± 0.9***^,###^	35.4 ± 0.1**^,##^
	GG-Hyl	20.0 ± 1.3	20.2 ± 0.6	10.2 ± 0.1***^,###^	10.6 ± 0.1***^,###^	20.1 ± 0.5
α2(I) K219	Lys	34.1 ± 0.5	34.3 ± 0.4	54.7 ± 0.5***^,###^	54.9 ± 0.2***^,###^	46.7 ± 0.2***^,###^
	Hyl	62.2 ± 0.7	62.0 ± 0.1	43.9 ± 0.5***^,###^	43.7 ± 0.3***^,###^	50.6 ± 0.3***^,###^
	G-Hyl	0.6 ± 0.3	0.7 ± 0.1	0.4 ± 0.1	0.3 ± 0.1	0.5 ± 0.0
	GG-Hyl	3.2 ± 0.4	3.0 ± 0.4	1.1 ± 0.1***^,##^	1.1 ± 0.1***^,###^	2.3 ± 0.0*
α1(I) K918/930	Lys + Lys	2.1 ± 0.1	1.3 ± 0.0	3.4 ± 0.2***^,###^	4.6 ± 0.2***^,###^	5.2 ± 0.1***^,###^
	Lys + Hyl	10.8 ± 0.1	9.5 ± 0.2	10.8 ± 0.1^###^	11.6 ± 0.1***^,###^	12.6 ± 0.0***^,###^
	Hyl + Hyl	87.1 ± 0.0	89.2 ± 0.2	85.8 ± 0.3***^,###^	83.8 ± 0.2***^,###^	82.2 ± 0.1***^,###^
α2(I) K933	Lys	0.3 ± 0.2	0.3 ± 0.2	1.3 ± 0.1***^,###^	1.2 ± 0.1***^,###^	0.7 ± 0.1*
	Hyl	99.7 ± 0.2	99.7 ± 0.2	98.7 ± 0.1***^,###^	98.8 ± 0.1***^,###^	99.3 ± 0.1*
α1(I) K9^N^	Lys	44.6 ± 0.5	48.0 ± 0.3	100 ± 0.0***^,###^	100 ± 0.0***^,###^	100 ± 0.0***^,###^
	Hyl	55.4 ± 0.5	52.0 ± 0.3	0.0 ± 0.0***^,###^	0.0 ± 0.0***^,###^	0.0 ± 0.0***^,###^
α1(I) K16^C^	Lys	51.9 ± 0.2	43.2 ± 0.3	100 ± 0.0***^,###^	100 ± 0.0***^,###^	100 ± 0.0***^,###^
	Hyl	48.1 ± 0.2	56.8 ± 0.3	0.0 ± 0.0***^,###^	0.0 ± 0.0***^,###^	0.0 ± 0.0***^,###^
α2(I) K5^N^	Lys	78.6 ± 0.8	77.3 ± 1.1	100 ± 0.0***^,###^	100 ± 0.0***^,##^	100 ± 0.0***^,###^
	Hyl	21.4 ± 0.8	22.7 ± 1.1	0.0 ± 0.0***^,###^	0.0 ± 0.0***^,##^	0.0 ± 0.0***^,###^

Lys hydroxylation and its glycosylation (%) represents the relative levels of Lys, Hyl, G-Hyl, and GG-Hyl (Lys + Hyl + G-Hyl + GG-Hyl = 100%).

Values represent mean ± S.D. (n = 3) of triplicate analysis for each group. Statistical differences were determined by Kruskal–Wallis one-way analysis of variance and means comparison with the controls by Dunnett’s method.

*Lys* lysine, *Hyl* hydroxylysine, *G-* galactosyl-, *GG-* glucosylgalactosyl, *MC* MC3T3-E1, *EV* empty vector, *KO* knock-out.

*p < 0.05, **p < 0.01, and ***p < 0.001 between MC and KO; ^##^*p* < 0.01 and ^###^*p* < 0.001 between EV and KO, respectively.

#### Lys hydroxylation in the telopeptides

**Figure 3:**
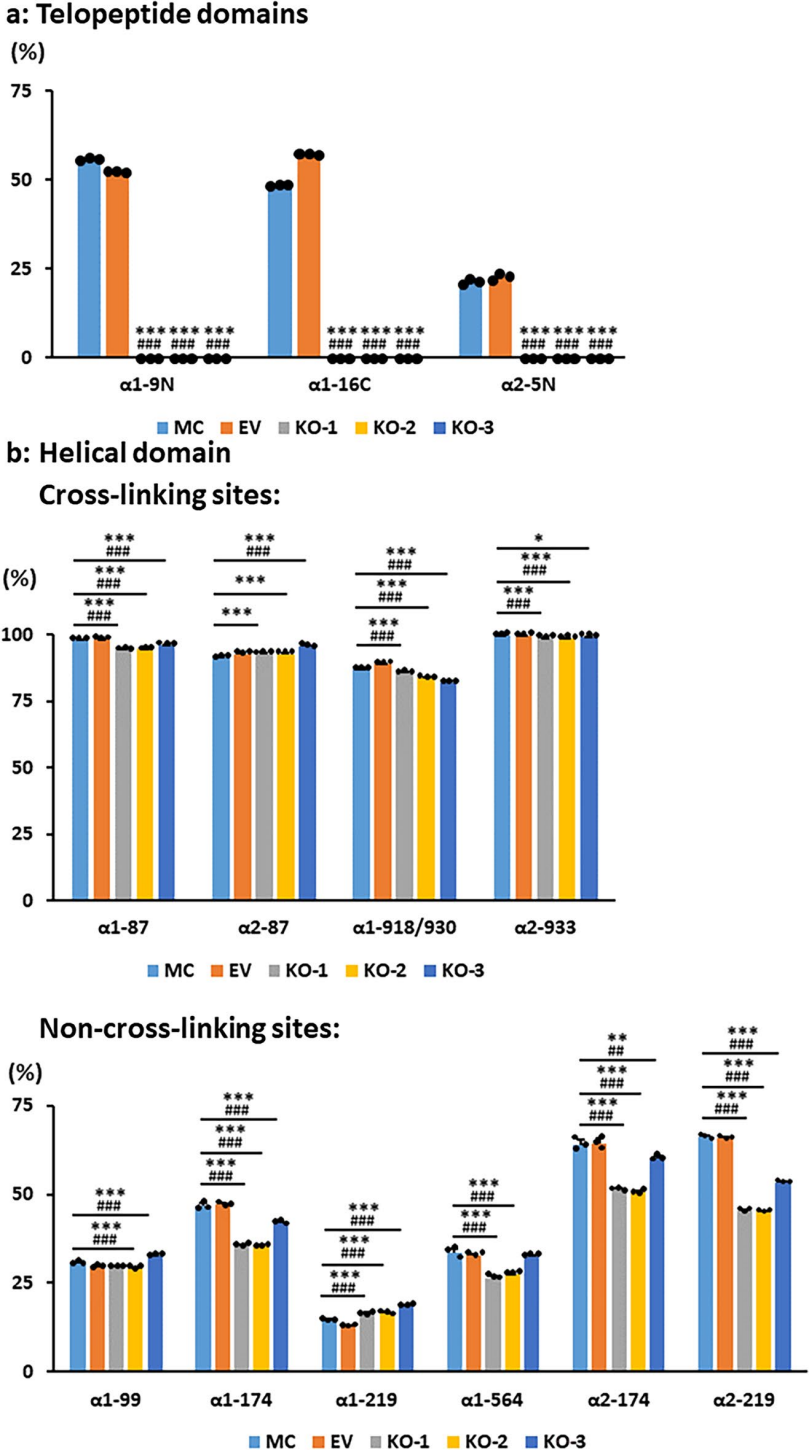
Extent of Lys hydroxylation at specific sites of type I collagen. (**a**) Lys hydroxylation in the telopeptides, (**b**) Lys hydroxylation in the cross-linking and non-cross-linking helical sites (see Table 2). Values represent percentages of Lys hydroxylation calculated as Hyl/(Lys + Hyl) × 100. Hyl in the helical domain is a sum of non-glycosylated, G, and GG-Hyl (see Table 3). *Lys* lysine, *Hyl* hydroxylysine, *G-* galactosyl-, *GG-* glucosylgalactosyl-, *MC* MC3T3-E1, *EV* empty vector, *KO* knock-out. Values represent mean ± S.D. (n = 3) of triplicate for each group. Statistical differences were determined by the method described above (Fig. 1 legend). *p < 0.05, **p < 0.01, and ***p < 0.001 between MC and KO; ^#^*p* < 0.05, ^##^*p* < 0.01, and ^###^*p* < 0.001 between EV and KO, respectively. The relative levels at α1(I)K918/930 show the percentage of “Hyl + Hyl”. See Table 3.

None of the telopeptidyl Hyl is glycosylated (Table 3). Lys hydroxylation in the telopeptides of type I collagen, i.e. N-telo (α1 Lys-9^N^ and α2 Lys-5^N^) and C-telo (α1 Lys-16^C^) (note: α2 C-telo lacks Lys), is shown in Fig. 3a. The values of MC and EV were essentially identical with no statistical difference, i.e. ~ 55.4% at α1 Lys-9^N^, ~ 22.7% at α2 Lys-5^N^ and ~ 56.8% at α1 Lys-16^C^ (Fig. 3a). In the KO type I collagen, however, none of the Lys residues was hydroxylated at any of these sites (Table 3, Fig. 3a). These results unequivocally demonstrate that LH2 is responsible for Lys hydroxylation in all telopeptides of type I collagen and that other LHs cannot compensate for this function.

#### Lys modifications in the helical domain

We then analyzed Lys modifications in the helical domain of type I collagen by using tryptic digests of collagen^8,33,37^ (Table 3, Fig. 3b). In the helical domain, modified Lys residues were identified at 11 sites. The values in Fig. 3 represent percentages calculated as [Hyl/(Hyl + Lys) × 100] where Hyl includes glycosylated (G- and GG-) and non-glycosylated forms (Table 3). First, we examined the helical cross-linking sites, i.e. α1 Lys-87, α1 Lys-930, α2 Lys-87 and α2 Lys-933. At α1 Lys-87, a highly hydroxylated and the most heavily glycosylated site of type I collagen^16,17^, ~ 98% of Lys was hydroxylated in controls, MC and EV. In KO collagen, it was also almost all hydroxylated, showing only 2–4% less hydroxylated than controls (Table 3). For α1 Lys-930, using the collagenase-pepsin digest^37^, we analyzed Lys hydroxylation in the peptide containing α1 Lys-918/930 (GDKGETGEQGDRGIKGHR). In controls, these Lys residues were at least 87–89% hydroxylated (Hyl + Hyl), and those in KO, at least 82–86% hydroxylated, again showing only a slight decrease of Lys hydroxylation. At α2 Lys-87, it was 92–93% in controls and 93–96% in KO indicating that Lys hydroxylation in KO at this site is almost the same level or even slightly higher than controls. The α2 Lys-933 was 99–100% hydroxylated in both controls and KO collagens. Thus, Lys hydroxylation was only minimally affected at the helical cross-linking sites in KO collagen. Second, we examined the helical non-cross-linking sites, i.e. α1 Lys-99, -174, -219, -564 and α2 Lys-174 and -219. For these sites the extent of Lys hydroxylation was almost the same at α1 Lys-99, slightly higher (1–4%) at α1 Lys-219, or up to ~ 20% lower at α1 Lys-174, -564, α2 Lys-174, α2 Lys-219 in KO type I collagen in comparison to controls (Fig. 3b). These data indicate that the contribution of LH2 towards helical Lys hydroxylation is low, especially at the cross-linking sites, and site-specific at non-crosslinking sites.

We next calculated the extent of glycosylation of Hyl at six sites identified, i.e., α1 Lys-87, α1 Lys-99, α1 Lys-174, α1 Lys-564, α2 Lys-174, and α2 Lys-219 (Table 4). When calculated as percentages of non-glycosylated-Hyl and glycosylated (G- and GG-) forms in total Hyl, the relative abundance of glycosylated Hyl at α1 Lys-87, the major glycosylation site, was slightly but significantly lower (2–10%) and non-glycosylated-Hyl significantly higher in KO collagen compared to those of controls (Table 4). At all other sites, i.e. α1 Lys-99, α1 Lys-174, α1 Lys-564, α2 Lys-174, and α2 Lys-219, the same phenomena, i.e. a lower level of glycosylation of Hyl in KO collagen, were observed (Table 4) with the exception of KO-3 exhibiting similar levels of non-glycosylated and glycosylated Hyl to controls at some sites (*p* > 0.05, respectively). These data indicate that LH2 deficiency may cause diminished glycosylation at several sites. Interestingly, when a percentage of two glycosylation forms (G- + GG- = 100%) was calculated, GG- form was lower and G- form higher at most sites in KO collagen when compared to controls (Table 5). These data suggest that LH2 deficiency causes a relative decrease of galactosylhydroxylysine-glucosyl transferase (GGT) activity leading to relative increase in the G-Hyl form, which is consistent with our recent report^6^.

**Table 4:** Glycosylation of hydroxylysine residues estimated by mass spectrometry of non-cross-linked glycosylated residues.

		Site occupancy (%)				
		MC	EV	KO-1	KO-2	KO-3
α1(I) K87	Hyl	4.0 ± 0.2	3.3 ± 0.1	13.0 ± 0.3***^,###^	12.6 ± 0.2***^,###^	5.3 ± 0.1***^,###^
	Glycosylated-Hyl	96.0 ± 0.2	96.7 ± 0.1	87.0 ± 0.3***^,###^	87.4 ± 0.2***^,###^	94.7 ± 0.1**^,###^
α1(I) K99	Hyl	65.3 ± 0.5	67.0 ± 0.6	74.5 ± 0.2***^,###^	73.5 ± 0.6***^,###^	76.5 ± 0.1***^,###^
	Glycosylated-Hyl	34.7 ± 0.5	33.0 ± 0.6	25.5 ± 0.2***^,###^	26.5 ± 0.6***^,###^	23.5 ± 0.1***^,###^
α1(I) K174	Hyl	88.3 ± 1.0	89.7 ± 0.4	92.7 ± 0.5***^,###^	92.2 ± 0.2***^,###^	91.0 ± 0.2***
	Glycosylated-Hyl	11.7 ± 1.0	10.3 ± 0.4	7.3 ± 0.5***^,###^	7.8 ± 0.2***^,###^	9.0 ± 0.2***
α1(I) K564	Hyl	72.3 ± 0.9	75.2 ± 0.2	84.3 ± 0.3***^,###^	83.8 ± 0.5***^,###^	84.2 ± 0.2***^,###^
	Glycosylated-Hyl	27.7 ± 0.9	24.8 ± 0.2	15.7 ± 0.3***^,###^	16.2 ± 0.5***^,###^	15.8 ± 0.2***^,###^
α2(I) K174	Hyl	9.7 ± 0.5	9.2 ± 0.7	15.6 ± 0.8***^,###^	16.0 ± 1.1***^,###^	7.7 ± 0.1*
	Glycosylated-Hyl	90.3 ± 0.5	90.8 ± 0.7	84.4 ± 0.8***^,###^	84.0 ± 1.1***^,###^	92.3 ± 0.1*
α2(I) K219	Hyl	94.3 ± 1.3	94.4 ± 0.8	96.8 ± 0.2**^,##^	96.9 ± 0.5**^,##^	94.9 ± 0.2
	Glycosylated-Hyl	5.7 ± 1.3	5.6 ± 0.8	3.2 ± 0.2**^,##^	3.1 ± 0.5**^,##^	5.1 ± 0.2

Glycosylation of Hyl residues (%) represents the relative levels of Glycosylated Hyl (G-Hyl + GG-Hyl). Hyl + Glycosylated Hyl = 100%.

Values represent mean ± S.D. (n = 3) of triplicate analysis for each group. Statistical differences were determined by Kruskal–Wallis one-way analysis of variance and means comparison with the controls by Dunnett’s method.

*Hyl* hydroxylysine, *G-* galactosyl-, *GG-* glucosylgalactosyl-, *MC* MC3T3-E1, *EV* empty vector, *KO* knock-out.

*p < 0.05, **p < 0.01, and ***p < 0.001 between MC and KO; ^##^*p* < 0.01 and ^###^*p* < 0.001 between EV and KO, respectively.

**Table 5:** Extent of two glycosylation forms of hydroxylysine in type I collagen isolated from MC and KO clone.

		Site occupancy (%)				
		MC	EV	KO-1	KO-2	KO-3
α1(I) K87	G-Hyl	8.2 ± 0.2	8.2 ± 0.2	12.8 ± 0.4***^,###^	12.6 ± 0.5***^,###^	8.0 ± 0.9
	GG-Hyl	91.8 ± 0.2	91.8 ± 0.2	87.2 ± 0.4***^,###^	87.4 ± 0.5***^,###^	92.0 ± 0.9
α1(I) K99	G-Hyl	64.9 ± 1.0	65.5 ± 0.5	73.2 ± 0.6***^,###^	72.3 ± 1.1***^,###^	63.9 ± 0.6
	GG-Hyl	35.1 ± 1.0	34.5 ± 0.8	26.8 ± 0.6***^,###^	27.7 ± 1.1***^,###^	36.1 ± 0.6
α1(I) K174	G-Hyl	56.8 ± 1.5	56.8 ± 2.2	73.0 ± 1.1***^,###^	71.5 ± 2.3***^,###^	65.8 ± 0.7***^,###^
	GG-Hyl	43.2 ± 1.5	43.2 ± 2.2	27.0 ± 1.1***^,###^	28.5 ± 2.3***^,###^	34.2 ± 0.7***^,###^
α1(I) K564	G-Hyl	47.1 ± 1.4	48.9 ± 2.6	63.0 ± 1.8***^,###^	64.0 ± 3.5***^,###^	58.2 ± 1.2***^,##^
	GG-Hyl	52.9 ± 1.4	51.1 ± 2.6	37.0 ± 1.8***^,###^	36.0 ± 3.5***^,###^	41.8 ± 1.2***^,##^
α2(I) K174	G-Hyl	65.3 ± 1.7	65.7 ± 0.5	76.5 ± 0.6***^,###^	75.1 ± 0.8***^,###^	63.8 ± 0.8
	GG-Hyl	34.7 ± 1.7	34.3 ± 0.5	23.5 ± 0.6***^,###^	24.9 ± 0.8***^,###^	36.2 ± 0.8
α2(I) K219	G-Hyl	14.8 ± 4.5	18.6 ± 0.5	27.4 ± 5.5*	23.5 ± 4.7	16.5 ± 1.8
	GG-Hyl	85.2 ± 4.5	81.4 ± 0.5	72.6 ± 5.5*	76.5 ± 4.7	83.5 ± 1.8

Glycosylation of Hyl residues (%) represents the relative levels of G-Hyl, and GG-Hyl (G-Hyl + GG-Hyl = 100%).

Values represent mean ± S.D. (n = 3) of triplicate analysis for each group. Statistical differences were determined by Kruskal–Wallis one-way analysis of variance and means comparison with the controls by Dunnett’s method.

*Hyl* hydroxylysine, *G-* galactosyl-, *GG-* glucosylgalactosyl-, *MC* MC3T3-E1, *EV* empty vector, *KO* knock-out.

*p < 0.01 and ***p < 0.001 between MC and KO; ^##^p < 0.01 and ^###^p < 0.001 between EV and KO, respectively.

### Pro 3-hydroxylation

Several sites of 3-hydroxyproline (3-Hyp), i.e. α1 Pro-986 and consecutive modification sites (α1 Pro-707, 716, 719 and α2 Pro-707, 716, 719)^38^ were identified (Table 6). In KO type I collagen, slight but significant increases of Pro 3-hydroxylation were observed at α1 Pro-986 (~ 91–93% for MC/EV, and ~ 94–98% for KO), and at α1/α2 Pro-707, 716 and 719, indicating that LH2 could be involved in this modification (Table 6).

**Table 6:** Summary of site-specific modification analysis by mass spectrometry of prolyl 3-hydroxylation in type I collagen from controls (MC and EV) and KO clones.

		Site occupancy (%)				
		MC	EV	KO-1	KO-2	KO-3
α1(I) [975–990]	0 × 3-Hyp	8.7 ± 0.2	6.8 ± 0.1	6.1 ± 0.0***^,###^	5.9 ± 0.1***^,###^	2.4 ± 0.1***^,###^
	1 × 3-Hyp	91.3 ± 0.2	93.2 ± 0.1	93.9 ± 0.0***^,###^	94.1 ± 0.1***^,###^	97.6 ± 0.1***^,###^
α1(I) [705–725]	0 × 3-Hyp	83.3 ± 1.2	84.4 ± 0.5	79.6 ± 0.4***^,###^	79.7 ± 0.3***^,###^	71.0 ± 0.1***^,###^
	1 × 3-Hyp	16.1 ± 0.8	14.9 ± 0.3	19.0 ± 0.3***^,###^	18.8 ± 0.3***^,###^	26.9 ± 0.1***^,###^
	2 × 3-Hyp	0.6 ± 0.4	0.7 ± 0.1	1.5 ± 0.1**^,#^	1.6 ± 0.0**^,##^	1.9 ± 0.0***^,###^
	3 × 3-Hyp	0.0 ± 0.0	0.0 ± 0.0	0.0 ± 0.0	0.0 ± 0.0	0.1 ± 0.0*^,#^
α2(I) [705–725]	0 × 3-Hyp	66.9 ± 0.4	67.4 ± 0.3	63.4 ± 0.2***^,###^	63.3 ± 0.6***^,###^	48.4 ± 0.6***^,###^
	1 × 3-Hyp	31.7 ± 0.1	31.4 ± 0.4	33.8 ± 0.1***^,###^	33.9 ± 0.5***^,###^	47.5 ± 0.5***^,###^
	2 × 3-Hyp	1.4 ± 0.5	1.3 ± 0.1	2.8 ± 0.1***^,###^	2.8 ± 0.1***^,###^	4.2 ± 0.2***^,###^
	3 × 3-Hyp	0.0 ± 0.0	0.0 ± 0.0	0.0 ± 0.0	0.0 ± 0.0	0.0 ± 0.0

Prolyl 3-hydroxylation (%) represents the relative levels of prolyl 3-hydroxylation at α1 Pro-986 (0–1 × 3-Hyp), α1 Pro-707, 716, 719 (0–3 × 3-Hyp) and α2 Pro-707, 716, 719 (0–3 × 3-Hyp) (total of 0–3 × 3-Hyp = 100%).

Values represent mean ± S.D. (n = 3) of triplicate for each group. Statistical differences were determined by Kruskal–Wallis one-way analysis of variance and means comparison with the controls by Dunnett’s method.

*Hyp* hydroxyproline, *MC* MC3T3-E1, *EV* empty vector, *KO* knock-out.

*p < 0.05, **p < 0.01, and ***p < 0.001 between MC and KO; ^#^*p* < 0.05, ^##^*p* < 0.01, and ^###^*p* < 0.001 between EV and KO, respectively.

### Collagen cross-link analysis

**Figure 4:**
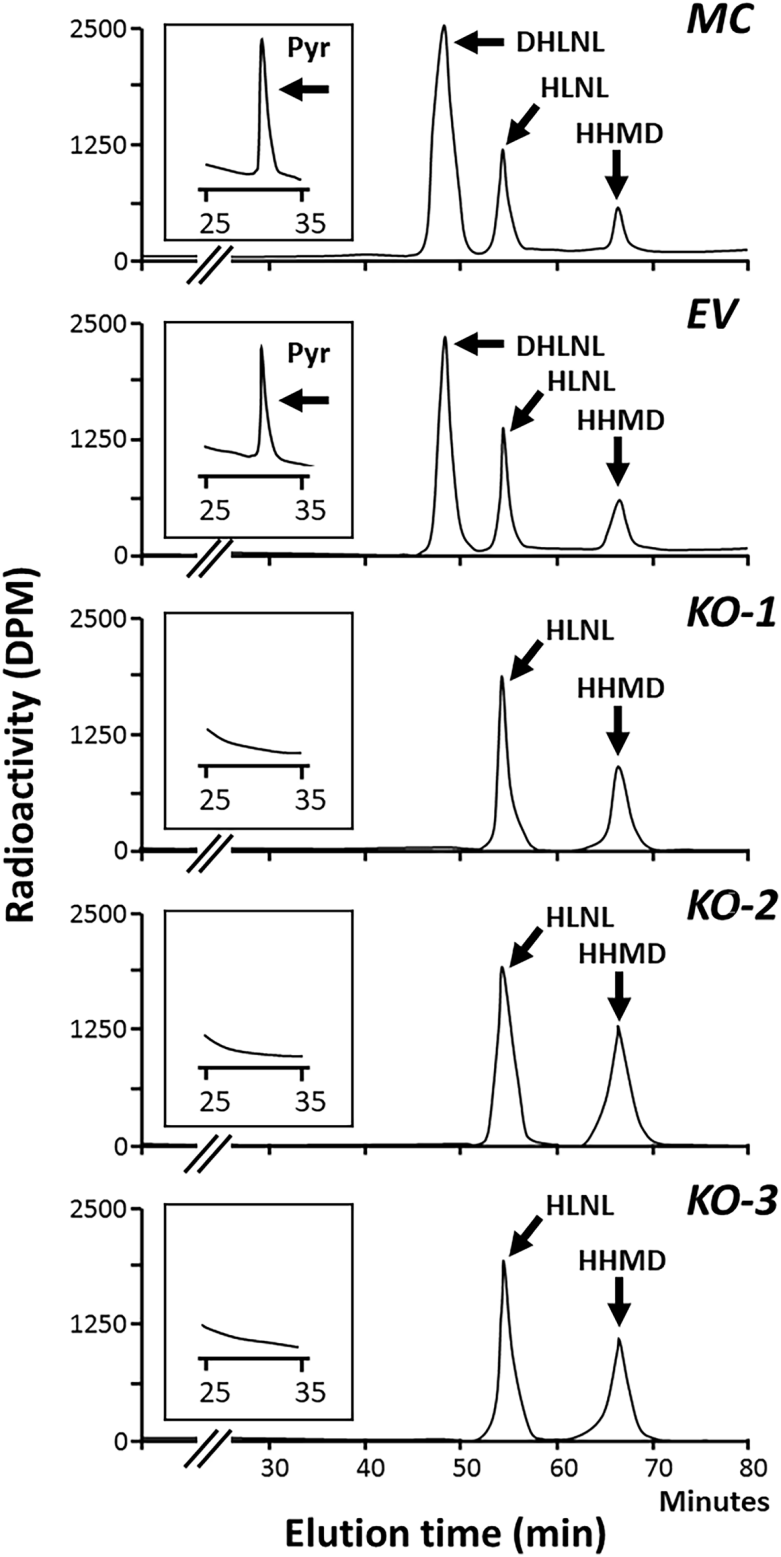
Typical chromatographic patterns of collagen cross-links from the acid hydrolysates of reduced collagen obtained from MC, EV, and KO clones. In MC and EV, cross-links were composed of DHLNL, HLNL, HHMD, and Pyr. However, in KO, there was a lack of DHLNL and Pyr. *DHLNL* dihydroxylysinonorleucine, *HLNL* hydroxylysinonorleucine, *HHMD* histidinohydroxymerodesmosine, *Pyr* pyridinoline, *MC* MC3T3-E1, *EV* empty vector, *KO* knock-out.

Control groups (MC and EV) showed essentially identical cross-link patterns (Fig. 4) with no statistical difference in any of the cross-links. The amounts of cross-links of control and KO collagens are summarized in Table 7. In control groups, the major cross-link was DHLNL (Hyl^ald^ × Hyl) representing ~ 67% of the total cross-links. The rest includes HLNL (Hyl^ald^ × Lys or Lys^ald^ × Hyl), Pyr (Hyl^ald^ × Hyl^ald^ × Hyl) and HHMD (Lys^ald^ × Lys^ald^ × His × Hyl). In KO collagen, none of the Hyl^ald^-derived cross-links (DHLNL, Pyr) were detected while Lys^ald^-derived cross-links, HLNL and HHMD, were both significantly increased by ~ 44 and ~ 400%, respectively. Though HLNL can be derived from Hyl^ald^ or Lys^ald^, since Lys at the helical cross-linking sites are almost fully hydroxylated and telopeptidyl Lys is not hydroxylated in KO collagen (Table 3), it should be derived from Lys^ald^ × Hyl in KO. In contrast to the striking difference in the type of cross-links, the difference in the total number of aldehydes involved in cross-linking is small (0.1–0.2 mol/mole of collagen) between control and KO collagens. This indicates that LOX/LOXL activities are not significantly affected in KO clones.

**Table 7:** Levels of immature reducible cross-links (DHLNL and HLNL) and mature non-reducible cross-links (Pry and HHMD) from MC, EV, and KO clones.

Cells/clones	DHLNL	HLNL	Pyr	HHMD	Total aldehydes
MC	0.74 (0.01)	0.27 (0.02)	0.021 (0.004)	0.08 (0.02)	1.21 (0.05)
EV	0.61 (0.05)	0.29 (0.01)	0.018 (0.001)	0.07 (0.01)	1.07 (0.02)
KO-1	ND	0.37 (0.01)***^,##^	ND	0.30 (0.02)***^,###^	0.98 (0.02)***
KO-2	ND	0.39 (0.01) ***^,###^	ND	0.29 (0.01)***^,###^	0.97 (0.03)***^,#^
KO-3	ND	0.36 (0.02)***^,##^	ND	0.28 (0.01)***^,###^	0.93 (0.02)***^,##^

Total aldehydes = DHLNL + HLNL + 2 × Pyr + 2 × HHMD. Values represent mean moles/mole collagen ± S.D. (n = 3) of triplicate analysis of the hydrolysates. Statistical differences were determined by Kruskal–Wallis one-way analysis of variance and means comparison with the controls by Dunnett’s method.

*DHLNL* dihydroxylysinonorleucine, *HLNL* hydroxylysinonorleucine, *HHMD* histidinohydroxymerodesmosine, *Pyr* pyridinoline, *MC* MC3T3-E1, *EV* empty vector, *KO* knock-out.

***p < 0.001 between MC and KO; ^#^*p* < 0.05, ^##^*p* < 0.01, and ^###^*p* < 0.001 between EV and KO, respectively.

### Collagen solubility, fibrillogenesis and matrix mineralization

**Figure 5:**
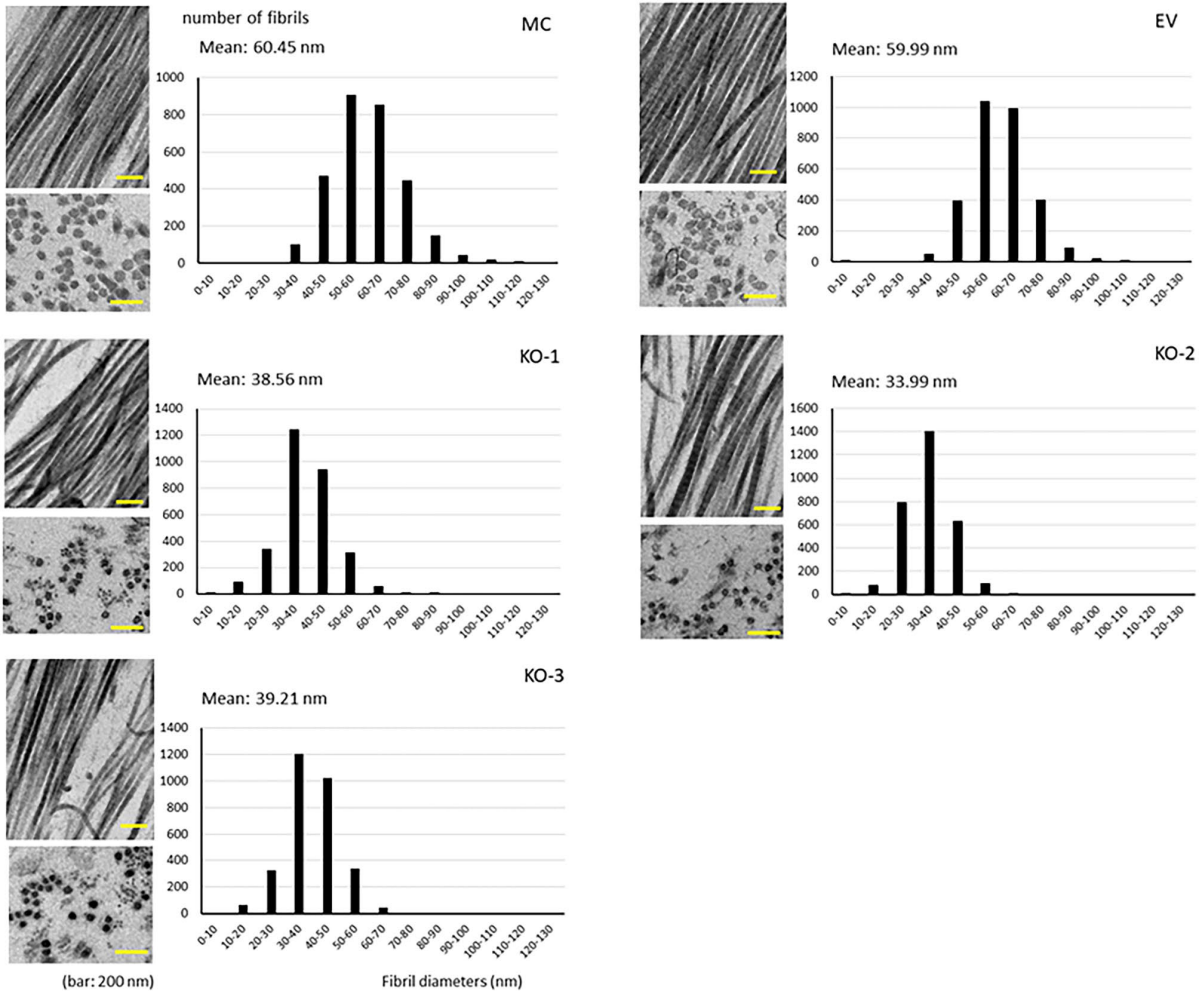
Ultrastructural analysis of collagen fibrils in cell cultures by transmission electron microscopy. The cross-sectional and longitudinal views of the collagen fibrils from MC, EV, and KO. KO collagen fibrils are markedly smaller in diameter. Scale bar represents 200 nm. Diameter distribution measured from cross-sections. Three thousand fibrils in each group were measured from a single experiment and plotted. *MC* MC3T3-E1, *EV* empty vector, *KO* knock-out.

**Figure 6:**
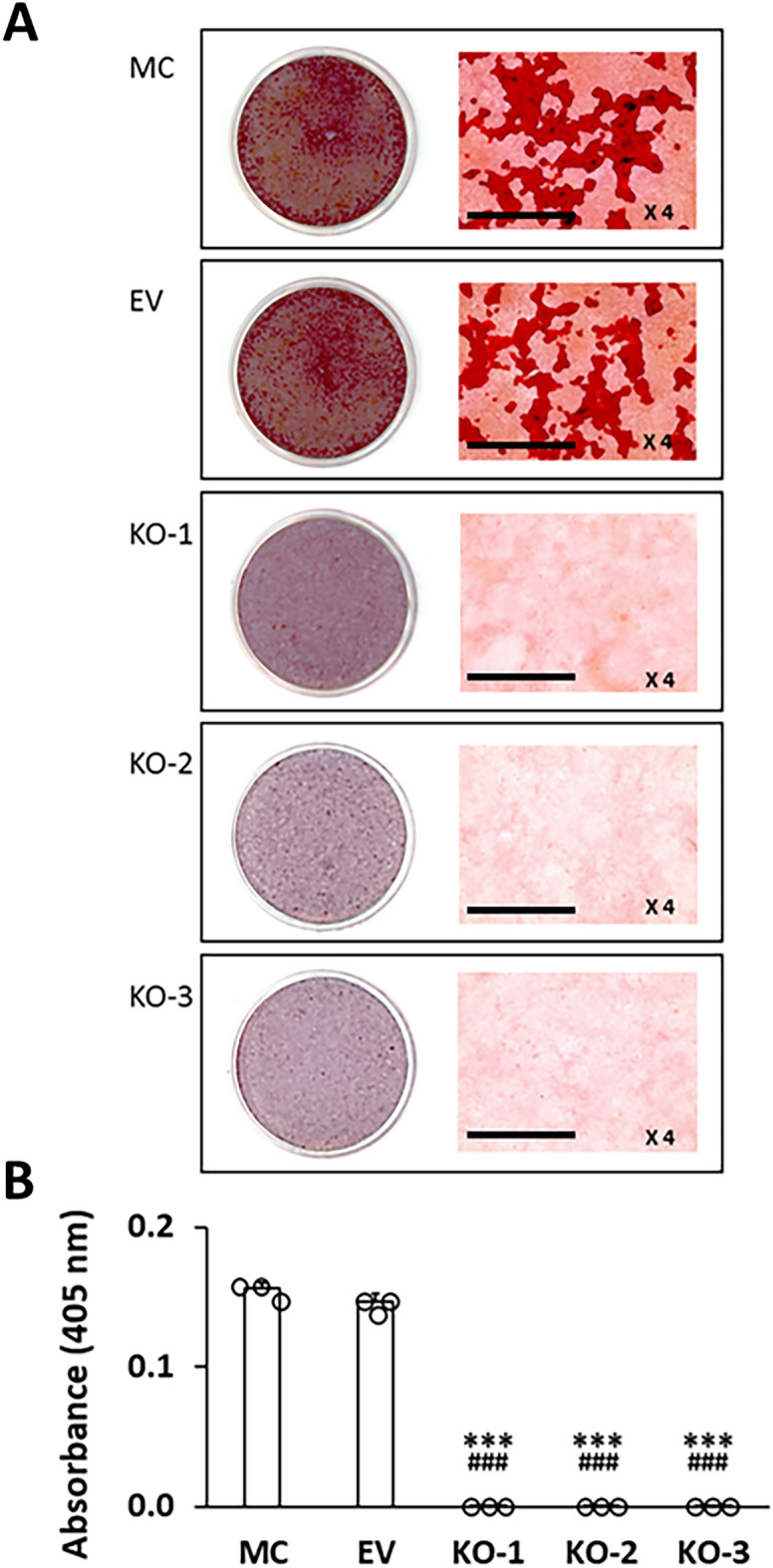
In vitro mineralization assay. (**A**) MC, EV, and KO clones were cultured in mineralization medium for 4 weeks. The cells/matrices were stained with Alizarin Red S. In the present study, an objective with a magnifying power of 4 was used to obtain the images. Scale bar represents 100 µm. (**B**) Quantification of Alizarin Red S contents. The contents were measured by absorbance at 405 nm. Values represent mean ± S.D. (n = 3) from three independent experiments. Statistical differences were determined by the method described above (Fig. 1. legend). ***p < 0.001 between MC and KO; ^###^*p* < 0.001 between EV and KO. *MC* MC3T3-E1, *EV* empty vector, *KO* knock-out.

We then evaluated the biochemical, morphological, and functional outcomes of LH2KO. First, we found that LH2KO resulted in a marked increase in collagen solubility (Table 8). Approximately 38% of KO collagen was solubilized with 0.5 M acetic acid while only trace amounts were solubilized in controls, MC (3.5%) and EV (2.7%) (*p* < 0.001). When the insoluble fractions with acetic acid (62.3–67.9% of KO and 96.5 and 97.3% of MC and EV collagens, respectively) were digested with pepsin, most of the KO collagen (53.7–61.6%) was solubilized while only 30.3 and 25.0% of collagen was solubilized in MC and EV, respectively (*p* < 0.001). After these serial extractions, the final insoluble collagen represented only 6.3–9.2% in KO collagen whereas the majority of collagen (66.2–72.4%) still remained insoluble in MC and EV (Table 8). These results clearly demonstrate that the lack of LH2-catalyzed modifications, i.e. primarily telopeptidyl Lys hydroxylation and subsequent cross-linking, makes collagen highly soluble. Second, we examined the effects of LH2KO on collagen fibrillogenesis. Representative cross-sectional/longitudinal views of collagen fibrils and the diameter distribution obtained from the cultures of controls (MC and EV) and KO clones (KO-1, -2, and -3) are shown in Fig. 5. The fibrils in KO clones were generally circular in shape and overall similar to those of MC and EV. However, the collagen fibril diameters in all KO clones were smaller than those of MC and EV (Fig. 5), indicating defective lateral growth of fibrils in KO collagen. Lastly, we assessed the effects of LH2KO on in vitro mineralization. The controls (MC and EV) and KO clones (1–3) were cultured for 28 days and subjected to mineralization assay using Alizarin red S staining (Fig. 6). In the controls (MC and EV), mineralized nodules were well formed at this point, however, no nodules were observed in KO clones at this time point (Fig. 6a,b), demonstrating that the lack of LH2 results in defective matrix mineralization.

**Table 8:** Solubility of collagen from MC, EV and KO clones.

	MC (%)	EV (%)	KO-1 (%)	KO-2 (%)	KO-3 (%)
Acetic acid	3.5 (0.2)	2.7 (0.1)	37.1 (0.2)***^,###^	37.7 (0.3)***^,###^	32.1 (0.1)***^,###^
Pepsin	30.3 (0.4)	25.0 (0.3)	53.7 (0.1)***^,###^	54.1 (0.3)***^,###^	61.6 (0.1)***^,###^
Residue	66.2 (0.6)	72.4 (0.4)	9.2 (0.1)***^,###^	8.2 (0.0)***^,###^	6.3 (0.1)***^,###^

The numbers represent percentage of total collagen sequentially extracted with 0.5 M acetic acid and pepsin, and final residues.

Values represent mean ± S.D. (n = 3) of triplicate analysis of collagen from MC, EC, and KO clones. Statistical differences were determined by Kruskal–Wallis one-way analysis of variance and means comparison with the controls by Dunnett’s method.

*MC* MC3T3-E1, *EV* empty vector, *KO* knock-out.

***p < 0.001 between MC and KO; ^###^*p* < 0.001 between EV and KO, respectively.

## Discussion

In this study, by generating LH2KO clones, we extensively characterized the molecular phenotypes of type I collagen. The lack of LH2 resulted in complete absence of Lys hydroxylation in all telopeptides, i.e. N- (9^N^) and C-telo (16^C^) of an α1 and N-telo (5^N^) of an α2 chains, thus, LH2 is solely responsible for hydroxylation of all Lys residues in telopeptides. Consistent with these data, the Hyl^ald^-derived cross-links, the major cross-links in MC/EV collagen, were completely absent from KO collagen and were replaced with Lys^ald^-derived cross-links. Moreover, our data indicated that LH2 may also be involved in helical Lys hydroxylation in a site-specific manner. The lack of LH2-catalyzed modification has significant impact on collagen solubility, collagen fibrillogenesis and matrix mineralization. In addition, LH2 could be involved in glucosylation of galactosyl Hyl. It should be noted, however, that this study was conducted using osteoblastic MC3T3 cells, thus, the phenotypes observed could be due in part to attributes of these cells.

Though the role of LH2 as telopeptidyl LH has been widely accepted^28^, the evidence reported thus far was not complete due mainly to the lack of appropriate models and analytical tools. Since LH2 KO mice die at early embryonic stage^32^, we generated LH2 KO clones using MC cells. MC cells are derived from normal mouse calvaria and collagen phenotypes are well-characterized^14,16,19,20,36^. MC cells synthesize predominantly type I collagen (> 96% of total collagen)^36,39^, Lys residues on type I collagen including cross-linking sites are only partially hydroxylated, all LHs (LH1-3) are well expressed, and collagen cross-links are sufficiently formed within 2 weeks of culture and mature with predictable kinetics^16,40^. These characteristics make MC cells an excellent model to investigate the biological functions of Lys modifications by manipulating specific LH gene expression and characterizing its effects on type I collagen^41^. Our current data unequivocally demonstrate that all Lys residues in telopeptides are hydroxylated solely by LH2, and neither LH1 nor LH3 can compensate for this function. It is not clear at this point what determines such substrate specificity for LH2. However, considering the fact that an acidic amino acid, Glu or Asp, is positioned next/close to telopeptidyl Lys residues (i.e. -Glu-Lys-Ser- in N- and C-telo of an α1 chain in both mouse and human, and -Asp-Lys-Gly- or -Asp-Gly-Lys-Gly- in N-telo of the mouse or human α2 chain, respectively), the presence of two basic Arg residues adjacent to the catalytic site of LH2 (R680 and R682) is likely important to determine such specificity^6^. Notably, these Arg residues are absent in LH1 or LH3 which explains their inability to compensate for LH2 deficiency. It is also interesting to note that, in MC/EV type I collagen, both N- and C-telo Lys residues of an α1 chain are ~ 50% hydroxylated while the N-telo Lys of an α2 chain is only ~ 20% hydroxylated. Possibly, the Asp-Lys-Gly- sequence of the latter that is also present in the helical domain may not be an optimal substrate for LH2. This is likely the reason why the Lys^ald^-involved cross-links are often derived from the α2 N-telo domain^17,42,43^.

Duran et al. has recently reported that a chaperone complex formed by Hsp47, FKBP65 and BiP modulates telopeptide Lys hydroxylation of type I procollagen chains. Defects of the complex members affected this modification either by enhancing (defect in Hsp47 and Bip) or diminishing (defect in FKBP65) LH2 activity^34^. In contrast, Syx et al. has stated that a mutant Hsp47, which showed a reduced binding to type I collagen, resulted in decreased LH2^44^. These inconsistent data suggest that Hsp47 may act as a positive or negative regulator of LH2 in a context-dependent manner. Interestingly, our present study showed that Fkbp65, Hsp47 and Bip protein levels were reduced in KO clones compared to MC (Fig. 2), suggesting that this chaperone complex may be destabilized by the lack of LH2.

It has been speculated that LH2 also catalyzes helical Lys hydroxylation based on its ability to hydroxylate the Lys residues in the synthetic (Ile-Lys-Gly)_3_ peptide and the data from the LH2/proα1(I) co-expression in an insect cell system^22^. The results indicate that, in this system, LH2 may function as a helical LH when LH1 and 3 are absent. However, the effect of LH2 expression on Lys hydroxylation at the specific molecular loci in an α1 chain including its C-telo domain or in an α2 chain including its N-telo domain were not investigated. Recently, Gistelinck et al. has reported that, in the bone from a 4-year old patient carrying a *PLOD2* heterozygous mutation, Lys in the α1(I) telopeptides was severely underhydroxylated while Lys at the helical cross-linking sites in type I collagen was normally hydroxylated^25^. Their findings are consistent with our current cell-based study showing that, when LH2 is absent, on the contrary to the changes in Lys hydroxylation in the telopetides, the extent of Lys hydroxylation in the helical domain was only minimally affected (Table 3, Fig. 3). It is important to note that, when these percentage differences are converted to the number of Hyl residues in a collagen molecule, the difference between MC/EV and KO is less than ± 0.03 residues at the cross-linking sites (α1–87, α1–918/930, α2–87, α2–933) and 0–0.2 residues at the non-cross-linking sites. Since Lys hydroxylation at the helical cross-linking sites is predominantly catalyzed by LH1 and its complex such as prolyl 3-hydroxylase 3 (P3H3), Synaptonemal Complex 65 (SC65) and CypB^9,33,45,46^, it is not surprising that absence of LH2 does not significantly affect Lys hydroxylation at these functionally critical sites in the helical domain. The significance of Lys hydroxylation at other sites in the helical domain is not well defined but, possibly, they may affect the interaction between collagen and collagen-binding proteins such as small leucine-rich proteoglycans and/or cell surface receptors such as integrins and discoidin domain receptor 2^47^.

Recently, Ishikawa and co-workers reported that the cooperation between LH1 and P3H3 is required for Lys hydroxylation in the helical domain of type I collagen, and that P3H3 may function as helical LH at specific cross-linking sites^46^. They also reported that LH2 level remained unchanged in LH1 null mice^46^. In the present study, we did not find a significant change of LH1 protein in LH2 KO clones. These findings suggest that there is no apparent direct interaction between LH1 and LH2. Thus, LH2 deficiency caused only a minute change in Lys hydroxylation in the helical domain of type I collagen.

One of the intriguing findings in the current study was that absence of LH2 affects Hyl glycosylation pattern. When the percentages of G- and GG- forms in total glycosylation forms (G- + GG-) are calculated, KO collagen showed that at most sites, the GG- was decreased at the expense of G- form in KO type I collagen (Table 5). Recently, we have reported that LH2 potentially has galactosylhydroxylysyl glucosyltransferase (GGT) activity^6^ and the current data (Tables 5, 9) supports this notion.

**Table 9:** Ratio of galactosyl (G)- to glucosylgalactosyl (GG)-hydroxylysine in type I collagen isolated from MC and KO clone.

	Ratio (G-Hyl/GG-Hyl)				
	MC	EV	KO-1	KO-2	KO-3
α1(I) K87	0.09 ± 0.00	0.09 ± 0.00	0.15 ± 0.01***^,###^	0.14 ± 0.01***^,###^	0.09 ± 0.01
α1(I) K99	1.85 ± 0.10	1.90 ± 0.07	2.73 ± 0.10***^,###^	2.61 ± 0.14***^,###^	1.77 ± 0.05
α1(I) K174	1.32 ± 0.10	1.32 ± 0.12	2.71 ± 0.18***^,###^	2.52 ± 0.28***^,###^	1.93 ± 0.06**^,##^
α1(I) K564	0.89 ± 0.06	0.96 ± 0.10	1.71 ± 0.17***^,###^	1.79 ± 0.27***^,###^	1.39 ± 0.07**^,#^
α2(I) K174	1.89 ± 0.17	1.92 ± 0.04	3.25 ± 0.13***^,###^	3.02 ± 0.12***^,###^	1.76 ± 0.06
α2(I) K219	0.18 ± 0.07	0.23 ± 0.01	0.39 ± 0.12*	0.31 ± 0.08	0.20 ± 0.02

Values represent ratio of Hyl glycosylation calculated as G-Hyl/GG-Hyl.

Values represent mean ± S.D. (n = 3) of triplicate for each group. Statistical differences were determined by Kruskal–Wallis one-way analysis of variance and means comparison with the controls by Dunnett’s method.

*Hyl* hydroxylysine, *G-* galactosyl-, *GG-* glucosylgalactosyl-, *MC* MC3T3-E1, *EV* empty vector, *KO* knock-out.

*p < 0.05, **p < 0.01, and ***p < 0.001 between MC and KO; ^#^*p* < 0.05, ^##^p < 0.01, and ^###^*p* < 0.001 between EV and KO (n = 3), respectively.

Prolyl 3-hydroxylation, another post-translational modification of collagen, is catalyzed by a complex composed of cartilage associated protein (CRTAP), P3H1 and CypB^48^. The deficiency of any of these components severely affects this modification leading to severe forms of recessive osteogenesis imperfecta^49–51^. It has been reported that the α1 Pro-986, the major site for this modification, is hydroxylated by P3H1^49^, and another modification site, α1/2 Pro-707, mainly by P3H2^52^. In the present study, we found that the extent of P3H at these sites was slightly increased in KO clones, suggesting that LH2 may interact with the P3H complex for prolyl-3-hydroxylation at these sites (Table 6). Since LH2 interacts with CypB^33^, a P3H complex member, these slight changes could occur by the lack of this interaction.

The impact of LH2 deficiency on collagen stability, fibrillogenesis and mineralization was striking. First, collagen solubility with dilute acid and pepsin digestion were markedly increased in KO collagen, i.e. > 90% of KO collagen was solubilized by these treatments while it was only ~ 30% in control groups. The marked increases in solubility in KO collagen can be explained by the differences in the nature of the cross-links. In KO collagen, since telopeptidyl Lys is not hydroxylated, the cross-links formed are all Lys^ald^-derived, aldimine cross-links such as deH-HLNL and deH-HHMD. The aldimine bond is known to be labile to dilute acids, thus, readily dissociated^53^. In contrast, the Hyl^ald^-derived bifunctional aldimine cross-links are spontaneously rearranged to ketoamines that are stable to dilute acids. The collagens containing the stable Hyl^ald^-derived cross-links are also more resistant against enzymatic degradation than those with the Lys^ald^-derived cross-links^54,55^. Since the total number of aldehydes involved in cross-linking is only ~ 8% lower in KO collagen compared to the control, the data implies that the Hyl^ald^-derived cross-linking is critical to confer insolubility on type I collagen. This is likely the reason why collagen enriched in the Hyl^ald^-derived cross-links accumulates without being readily degraded by proteolytic enzymes in fibrosis^28,56,57^ and also in desmoplastic tumors such as pancreatic ductal adenocarcinoma^58^, lung cancer^29^, breast cancer^31,59^ and oral cancer^30^. Such stiffened collagen matrix may not only form a shelter for cancer cells to protect them from immune cells and anti-cancer drugs but also serve as a means for cancer cells to attach, migrate and metastasize efficiently^41,60,61^. Second, based on one experiment, fibrillogenesis in LH2 KO collagen is also affected showing smaller fibril diameters compared to those of controls. This could be due to several factors including: 1. since KO collagen is more susceptible to degradation (see above), collagen fibrils may not be able to grow, 2. altered Lys modifications (hydroxylation and glycosylation) of KO collagen may favor the association with collagen-binding proteins, such as decorin, that is known to inhibit collagen fibrillogenesis^62–64^, 3. altered post-translational modifications in KO collagen may inherently limit the growth of molecular packing into a fibril. Notably, when LH2 is overexpressed in MC cells, collagen fibrils are also smaller than controls^20^. This may indicate that the extent of LH2-mediated post-translational modifications should be kept at a certain range to establish an appropriate size of collagen fibrils in this cell culture system.

In bone, fibrillar type I collagen functions as an organizer of mineral deposition and growth^65–67^. Since initial mineralization appears to occur in the intermolecular channel formed by contiguous hole zones in the collagen fibril^68^, the pattern of intermolecular cross-linking formed at the edge of hole zones should be critical to organize mineralization^69^. The LH2 KO collagen fibrils that contain abnormal cross-linking, highly soluble and smaller in size may not serve well as a stable template to accommodate and organize matrix mineralization. This may result in defective bone formation as seen in Bruck syndrome 1 and 2, that are caused by mutations in genes encoding LH2 chaperone FKBP65 and LH2, respectively. In addition to the structural function, LH2 may regulate cellular activities through its action on integrin β1^70^ that may also impact the mineralization process.

Recently, we have reported bone phenotypes of LH2 heterozygous mice (LH2^+/−^) in which LH2 expression levels are only ~ 50% of those of wild type mice (LH2^+/+^). In this animal model, LH2^+/−^ femurs showed lower bone mineral density and inferior bone mechanical properties compared to those of LH2^+/+^ mice^71^. When cultured, LH2^+/−^ osteoblastic cells mineralized poorly, which is consistent with our current study. Thus, while we cannot determine to what extent the LH2-catalyzed modification directly contributes to collagen mineralization, such modification appears to play a critical role in this process.

LH2 has two isoforms: one with an additional 63 bp-exon 13A (LH2b) and the other without (LH2a)^5^. It is generally accepted that LH2b is the telopeptidyl LH, but recently it has been reported that LH2a is also capable of catalyzing Lys hydroxylation in the telopeptides^6^. Inducing these isoforms in the LH2 KO cells separately and characterizing their collagen molecular phenotypes will provide valuable insights into their distinct and/or overlapping functions. This is now underway in our laboratory and will be the subject of the separate publication.

In conclusion, this study demonstrates that the major function of LH2 is to hydroxylate the N- (α1 and α2 chains) and C-telopeptidyl (α1 chain) Lys residues of type I collagen. The deficiency of LH2 profoundly affects collagen cross-linking, solubility, fibrillogenesis, and mineralization. These results underscore the pivotal role of the LH2-mediated post-translational modifications in the formation and function of fibrillar collagen in bone.

## Methods

### Cell lines and culture conditions

MC3T3-E1 subclone 4, a well characterized nontransformed mouse osteoblast-like cell line^72^, was purchased from American Type Culture Collection (CRL-2593). Cells were grown in α-minimum essential media (Invitrogen, Carlsbad, CA, USA) containing 10% FBS (Invitrogen) and supplemented with 100 units/ml penicillin G sodium and 100 μg/ml streptomycin sulfate in a 5% CO_2_ atmosphere at 37 °C. The medium was changed twice a week.

### Generation of MC cells lacking LH2 by CRISPR/Cas9n gene editing

To generate LH2 deficient (KO) cells, we used double-nicking strategy to minimize off-target mutagenesis^73^. One pair of gRNAs (gRNA 1 [antisense: ctcctccgccacgcccaggc] and 2 [sense: acgcccgggcgcatccctgc]) were chosen to target the exon 1 of the mouse *Plod2* gene (Supplementary Fig. S4). Oligonucleotide pairs containing these gRNA sequences were cloned into pX335 (Addgene) that contains D10A mutant Cas9 (Cas9n)^74^, to produce pX335-mLH2-1 and -2. The sequence-verified pairs of pX335-mLH2-1 and -2 (Eton Bioscience, Durham, NC, USA), together with a puromycin-containing plasmid were transfected into MC cells using FuGENE 6 transfection reagent (Roche Applied Sciences). The non-transfected MC cells and those transfected with the original pX335 plasmid (empty vector (EV); ligation of pX335 alone without annealed sgRNA oligo inert) were used as controls. After 48 h, the transfected cells were trypsinized, single-cell sorted into 96-well plates by fluorescence-activated cell sorter (FACS), and maintained in α-minimum essential medium, 10% FBS, 100 units/ml penicillin, 100 μg/ml streptomycin, and 2 μg/ml Puromycin (InvivoGen, San Diego, CA, USA). The expanded cells were characterized by sequencing the targeted region of *Plod2* gene and by comparing the level of LH2 with those of the EV and the non-transfected MC cells.

#### Evaluation of off-target effect

The specificity of the various gRNAs used in this study and their potential off-target cleavage probabilities were initially evaluated using two different algorithms, online CRISPR RGEN Tools and Off-Spotter design prior to deploying them in MC cells. We also used the *Plod2* sg RNAs as queries to search for similar sequences in the mouse genome using Cas-OFFinder (http://www.rgenome.net/cas-offinder/). To test whether *Plod2* sgRNAs target other genomic loci by mispairing, we performed experimental testing of five candidates selected from a search allowing up to two mismatches and two bulges. DNA primers were designed to amplify the DNA sequences containing the potential off-target sites in KO-1 using the standard PCR method (Supplementary Table S1). PCR products were separated by gel electrophoresis, and the DNA bands were isolated and extracted for Sanger sequencing. Sequencing results were aligned with mouse genome using blastn algorithm to identify potential sequence variants.

### Quantitative real-time PCR

To determine the expression of *Plod2*, MC, EV and KO clones were plated at a density of 2 × 10^5^ cells/35 mm-dish. After 48 h, total RNA was extracted with TRIzol reagent (Invitrogen). Expression levels of *Plod2* mRNA were assessed by one-step quantitative reverse transcription polymerase chain reaction (RT-PCR) with ABI Prism 7500 (Applied Biosystems). The specific probe and primers set for *Plod2* was purchased from ThermoFisher Scientific (TaqMan Gene Expression Assay, Mm00478767_m1) that amplifies exon 4–5 boundary, thus, amplifying both *Plod2a* and *Plod2b*. The mRNA expression levels were normalized to beta-actin (*Actb*; Mm01205647_g1) and analyzed by the 2^−ΔΔCT^ method^75^.

### Western blot analysis

To determine the protein level, the KO clones and controls were plated onto 35-mm dishes at a density of 3 × 10^5^ cells/dish. After culturing for 7 days, the cells were washed with phosphate-buffered saline (PBS), lysed with radio-immunoprecipitation assay (RIPA) lysis buffer (50 mM Tris–HCl, 150 mM NaCl, 0.5% Sodium deoxycholate, 0.1% SDS, and 1% NP-40), centrifuged at 12,000×*g* and the supernatant was collected. The total protein concentration was measured by the Pierce BCA Protein Assay Kit (Pierce Biotechnology, Rockford, IL, USA) according to the manufacturer’s protocol. The cell lysate was mixed with 2× Laemmli Sample Buffer containing 2-mercaptoethanol (BIO-RAD) and 10 µg of total protein was applied to a 4–20% Mini-PROTEAN TGX Precast Protein Gel (BIO-RAD). The separated proteins were transferred to a polyvinylidene fluoride (PVDF) membrane (Immobilon-P, Millipore Corp., Bedford, MA, USA) and probed with rabbit polyclonal anti-LH2 antibody (Proteintech Group, Inc., Rosemont, IL, USA). Other protein levels were also characterized using rabbit polyclonal PLOD1 antibody (1:200, cat# 12475-1-AP, Proteintech), rabbit polyclonal PLOD2 antibody (1:100, cat# 21214-1-AP, Proteintech) that recognizes both LH2b and LH2a^6^, rabbit polyclonal PLOD3 antibody (1:200, cat# 11027-1-AP, Proteintech), rabbit polyclonal GLT25D1 antibody (1:200, cat# 16768-1-AP, Proteintech), rabbit polyclonal Fkbp65 antibody (1:200, cat# 12172-1-AP, Proteintech), rabbit polyclonal CypB antibody (1:10,000, cat# PA1-027A, Thermo Fisher), rabbit polyclonal Hsp47 antibody (1:100, cat# 10875-1-AP, Proteintech), and rabbit polyclonal Bip antibody (1:100, cat# 11587-1-AP, Proteintech). Horseradish peroxidase (HRP)-conjugated anti-rabbit IgG (Cell Signaling Technology) was used as a secondary antibody and HRP-conjugated anti-β-actin rabbit monoclonal antibody (13E5, Cell Signaling Technology) was used as an internal control for protein loading. The reactivities of HRP were detected with SuperSignal West Pico Chemiluminescent Substrate (Thermo Fisher Scientific) and the chemiluminescence was scanned using an Odyssey Infrared Imaging System (LI-COR Biosciences). Quantitation of proteins was performed using the Image Studio software version 4.0 (LI-COR) with normalization to β-actin levels and was then shown as the change relative to the protein levels in MC as 1.0.

### Collagen preparation for biochemical analysis

MC, KO and EV clones were cultured in α-minimum essential media (Invitrogen) containing 10% FBS, 100 units/ml penicillin, and 100 μg/ml streptomycin. When the cells grew to confluence, the medium was replaced with that containing 50 μg/ml of ascorbic acid. After 2 weeks of culture, the cells/matrix layers were scraped, thoroughly washed with PBS and cold distilled water several times by repeated centrifugation at 4000×*g*, and lyophilized.

### Collagen type analysis

Collagen was extracted and purified from lyophilized cell/matrix layer of MC, EV, and KO clones by digestion with pepsin (Sigma-Aldrich, St. Louis, MO, USA; 5 mg/ml in 0.5 M acetic acid) and salt precipitation (0.7 M NaCl in 0.5 M acetic acid) as described previously^36^. Type I and III collagens were quantified by LC–MS using SI-collagen as an internal standard^35^. In brief, SI-collagen was first mixed into the purified collagen samples, and the samples were digested with sequencing grade trypsin (Promega, Madison, WI, USA; 1:50 enzyme/substrate ratio) in 100 mM Tris–HCl/1 mM CaCl_2_ (pH 7.6) at 37 °C for 16 h after heat denaturation at 60 °C for 30 min. Generated marker peptides of type I and III collagens (two peptides for each α chain; stable isotopically heavy and light ones) were monitored by LC-QqQ-MS on a 3200 QTRAP hybrid QqQ/linear ion trap mass spectrometer (AB Sciex, Foster City, CA, USA) with an Agilent 1200 Series HPLC system (Agilent Technologies, Palo Alto, CA, USA) using a BIOshell A160 Peptide C18 HPLC column (5 µm particle size, L × I.D. 150 mm × 2.1 mm; Supelco, Bellefonte, PA, USA) to determine the concentrations of type I and type III collagens.

### Reduction with NaB^3^H_4_

Lyophilized cell/matrix samples (~ 2.0 mg each) were suspended in buffer containing 0.15 M N-trismethyl-2-aminoethanesulfonic acid, and 0.05 M Tris–HCl, pH 7.4, and reduced with standardized NaB^3^H_4_. The specific activity of the NaB^3^H_4_ was determined by the method previously reported^76^. The reduced samples were washed with cold distilled water several times by repeated centrifugation at 4000×*g* and lyophilized.

### Quantification of Hyl by HPLC

Reduced collagen was hydrolyzed with 6 N HCl and subjected to amino acid analysis^77^. The level of total Hyl in a collagen molecule was calculated based on the value of 300 residues of Hyp per collagen molecule, which were quantified as residues/collagen molecule^14^.

### Site-specific characterization of post-translational modifications of type I collagen

The purified collagen samples were digested with trypsin as described above to analyze the Lys post-translational modifications at the specific molecular sites within the triple helical domain of type I collagen^37^. In addition, to analyze Lys hydroxylation at the telopeptide domains of type I collagen, the lyophilized cell/matrix samples were sequentially digested with bacterial collagenase and pepsin as previously reported^37^. In brief, the samples were digested with 0.01 mg/ml of collagenase from *Grimontia hollisae* (Nippi, Tokyo, Japan)^78^ in 100 mM Tris–HCl/5 mM CaCl_2_ (pH 7.5) at 37 °C for 16 h after heating at 60 °C for 30 min. After addition of acetic acid (final 0.5 M), the collagenase-digests were further digested with 0.01 mg/ml of pepsin (Sigma-Aldrich) at 37 °C for 16 h. The trypsin- or collagenase/pepsin-digests were subjected to LC-QTOF-MS analysis on an ultra-high resolution QTOF mass spectrometer (maXis II, Bruker Daltonics, Bremen, Germany) coupled to a Shimadzu Prominence UFLC-XR system (Shimadzu, Kyoto, Japan) using an Ascentis Express C18 HPLC column (5 µm particle size, L × I.D. 150 mm × 2.1 mm; Supelco)^37^. Site occupancy of Lys hydroxylation/glycosylation (Lys, Hyl, G-Hyl, and GG-Hyl) was calculated using the peak area ratio of extracted ion chromatograms (mass precision range = ± 0.05) of peptides containing the respective molecular species as previously reported^8,33,37,79^.

### Collagen cross-link analysis

Reduced collagen was hydrolyzed with 6 N HCl, and subjected to cross-link analysis as described previously^77^. Upon reduction, the dehydrodihydroxylysinonorleucine (dehydro-DHLNL)/its ketoamine, dehydrohydroxylysinonorleucine (dehydro-HLNL)/its ketoamine, and dehydrohistidinohydroxymerodesmosine (dehydro-HHMD) are reduced to stable secondary amines, DHLNL, HLNL, and HHMD. The reducible cross-links were analyzed as their reduced forms (i.e. DHLNL, HLNL, and HHMD, respectively). Hereafter, the terms DHLNL, HLNL, and HHMD will be used for both the unreduced and reduced forms. The levels of the major immature reducible, DHLNL, HLNL, and HHMD, and mature non-reducible cross-links, Pyr, were quantified as moles/mole of collagen^69,77^.

### Solubility of collagen

Solubility of collagen from lyophilized cell/matrix samples were evaluated by sequential extraction using acetic acid and pepsin as described previously with slight modification^79^. In brief, collagen was first extracted using 0.5 M acetic acid at 4 °C for 24 h, and subsequently extracted with 5 mg/ml high-purity pepsin (1:60,000; Wako Chemicals, Osaka, Japan) in 0.5 M acetic acid at 4 °C for 24 h. The acid- and pepsin-soluble fractions and the residual fraction were subjected to acid hydrolysis (6 N HCl, 110 °C for 20 h in the gas phase under N_2_) after addition of SI-collagen as an internal standard^35^. The acid hydrolysates were subjected to LC–MS analysis of 4-Hyp in MRM mode on the QqQ mass spectrometer using a ZIC-HILIC column (3.5 µm particle size, L × I.D. 150 mm × 2.1 mm; Merck Millipore, Billerica, MA, USA)^79^. Concentration of collagen was estimated by the peak area ratio of 4-Hyp to stable isotopically heavy 4-Hyp derived from SI-collagen.

### Measurements of collagen fibril diameter by transmission electron microscopy

MC, KO and EV clones were plated at a density of 2 × 10^5^ cells/35-mm dishes and cultured in α-minimum essential medium, 10% FBS, 100 units/ml penicillin, 100 μg/ml streptomycin, 50 μg/ml ascorbic acid, and 2 mM β-glycerophosphate, for 2 weeks. The cell/matrix layers were washed with PBS, fixed with 2.5% EM grade glutaraldehyde in 0.1 M sodium cacodylate buffer, pH 7.4. The samples were then postfixed in potassium ferrocyanide-reduced osmium for 1 h at room temperature. After rinsing with distilled water, the samples were dehydrated with a graded series of ethanol concentrations, and embedded in PolyBed-812 epoxy resin (Polysciences, Warrington, PA, USA). Sections of 70 nm thickness were cut, mounted on copper Formvar-carbon filmed grids, and stained with 4% uranyl acetate and Reynolds’ lead citrate^80^. Cross-sectional views of the collagen fibrils were observed using a LEO EM-910 transmission electron microscope operating at 80 kV (Carl Zeiss SMT, Peabody, MA, USA), and images were taken at 25,000× using a Gatan Orius SC1000 CCD camera with Digital Micrograph 3.11.0 (Gatan, Inc., Pleasanton, CA, USA). For each sample, the diameters of 3,000 fibrils were measured using ImageJ 1.44p software.

### In vitro mineralization assay

MC, EV and KO clones were plated at a density of 2 × 10^5^ cells/35-mm dish and cultured in α-minimum essential medium containing 10% FBS, 100 units/ml penicillin, and 100 μg/ml streptomycin. Upon confluence, cells were maintained in the mineralization medium containing 50 μg/ml ascorbic acid and 2 mM β-glycerophosphate and cultured for up to 4 weeks. The cell/matrix layer from each sample was washed with PBS, fixed with 100% methanol, and stained with 1% Alizarin Red S (Sigma Chemical, St. Louis, MO, USA). Then, the extent of mineralization was evaluated from the measurements of Alizarin Red S content by using the previous reported method^81^.

### Statistical analyses

Statistical analyses were performed using Jmp®8.0 software (SAS Institute Inc., Cary, NC, USA). Statistical differences were determined by Kruskal–Wallis one-way analysis of variance and means comparison with the controls by Dunnett’s method. The data were presented as means ± standard deviation (S.D.), and a *p* value less than 0.05 was considered to be statistically significant.

## Supplementary Information

Supplementary Information.

## Data Availability

All data are contained within this manuscript and supporting information. The MS data sets for specific lysine post-translational modification in type I collagen have been deposited to the Zenodo repository (https://zenodo.org/record/5211220#.YRxpjOjniUk). The all source data are available from the corresponding author upon request.
