# Supplementary material for: Lysyl hydroxylase 2 mediated collagen post-translational modifications and functional outcomes
Source: Sci Rep. 2022 Aug 22;12:14256. doi: 10.1038/s41598-022-18165-0 (PMC9395344; doi:10.1038/s41598-022-18165-0)
Supplement: Supplementary file 1 — Supplementary Information. [file 41598_2022_18165_MOESM1_ESM.docx]

Lysyl hydroxylase 2 mediated collagen post-translational modifications and functional outcomes

Masahiko Terajima^1¶^, Yuki Taga^2¶^, Tomoyuki Nakamura^3^, Hou-Fu Guo^4^, Yukako Kayashima^5^, Nobuyo Maeda-Smithies^5^, Kshitij Parag-Sharma^6^, Jeong Seon Kim^4^, Antonio L. Amelio^1, 7, 8, 9^, Kazunori Mizuno^2^, Jonathan M. Kurie^4^, Mitsuo Yamauchi^1^*

^1^Division of Oral and Craniofacial health Sciences, Adams School of Dentistry, University of North Carolina at Chapel Hill, North Carolina, United States of America

^2^Nippi Research Institute of Biomatrix, Ibaraki, Japan

^3^Department of Pharmacology, Kansai Medical University, Osaka, Japan

^4^Department of Molecular and Cellular Biochemistry, University of Kentucky, Kentucky, United States of America

^5^Department of Pathology and Laboratory Medicine, University of North Carolina at Chapel Hill, North Carolina, United States of America

^6^Graduate Curriculum in Cell Biology & Physiology, Biological & Biomedical Sciences Program, UNC School of Medicine, University of North Carolina at Chapel Hill, North Carolina, United States of America

^7^Department of Cell Biology and Physiology, UNC School of Medicine, University of North Carolina at Chapel Hill, North Carolina, United States of America

^8^Biomedical Research Imaging Center, UNC School of Medicine, University of North Carolina at Chapel Hill, North Carolina, United States of America

^9^Lineberger Comprehensive Cancer Center, Cancer Cell Biology Program, UNC School of Medicine, University of North Carolina at Chapel Hill, North Carolina, United States of America

^10^Department of Thoracic/Head and Neck Medical Oncology, University of Texas MD Anderson Cancer Center, Texas, United States of America

^¶^ These authors contributed equally to this work.

* To whom correspondence should be addressed: Mitsuo Yamauchi; 385 S Columbia Street, Koury Oral Health Sciences, Room 4606, Chapel Hill, NC 27514; Tel.: 919-537-3217; Fax: 919-966-3683; E-mail: [mitsuo_yamauchi@unc.edu](mailto:mitsuo_yamauchi@unc.edu).

**Supplementary Table S1.** Potential off-target sites from LH2 KO clones (KO-1) for Sanger Sequencing.

| Site | Potential off-target site | Chromosome | Strand | Position | Mismatches | Bulge size | Bulge type | Forward primer | Reverse primer | Amplicon length (bp) |
| --- | --- | --- | --- | --- | --- | --- | --- | --- | --- | --- |
| 1 | CTGCCTCCGCCAaaCCCAGGC-TGG | 8 | - | 33600286 | 2 | 1 | DNA | 5'-GTAACGTGGCCGGTTCTAAA-3' | 5'-CGAGTGTAAGCAGCTGAACG-3' | 485 |
| 2 | CTCCTCaGCCA-GCCCtGGC-TGG | 15 | + | 75828156 | 2 | 1 | RNA | 5'-GTGCAGCTAAACCTGCCTCT-3' | 5'-TCCCTCTGGTCAAGGACACT-3' | 217 |
| 3 | CTCCTCCGCGCACtCCCAGGg-AGG | 5 | - | 113844292 | 2 | 1 | DNA | 5'-AGTCAAGACGAGCGCATTTC-3' | 5'-CTTTCGGTTTTGGCAACATT-3' | 427 |
| 4 | CTCCTCCGCCTgCGCCCAGGg-CGG | 5 | - | 28317374 | 2 | 1 | DNA | 5'-GCACGAAGGCCCTACATAAA-3' | 5'-TCTGTGCGCTGCTCTTTCTA-3' | 421 |
| 5 | CTCCTCC-CCACaCCCAGcC-TGG | 5 | + | 119243707 | 2 | 1 | RNA | 5'-TGACCTGGGCACCATTTATT-3' | 5'-TCTGGGAAGCATCCTCT-3' | 456 |

**a**


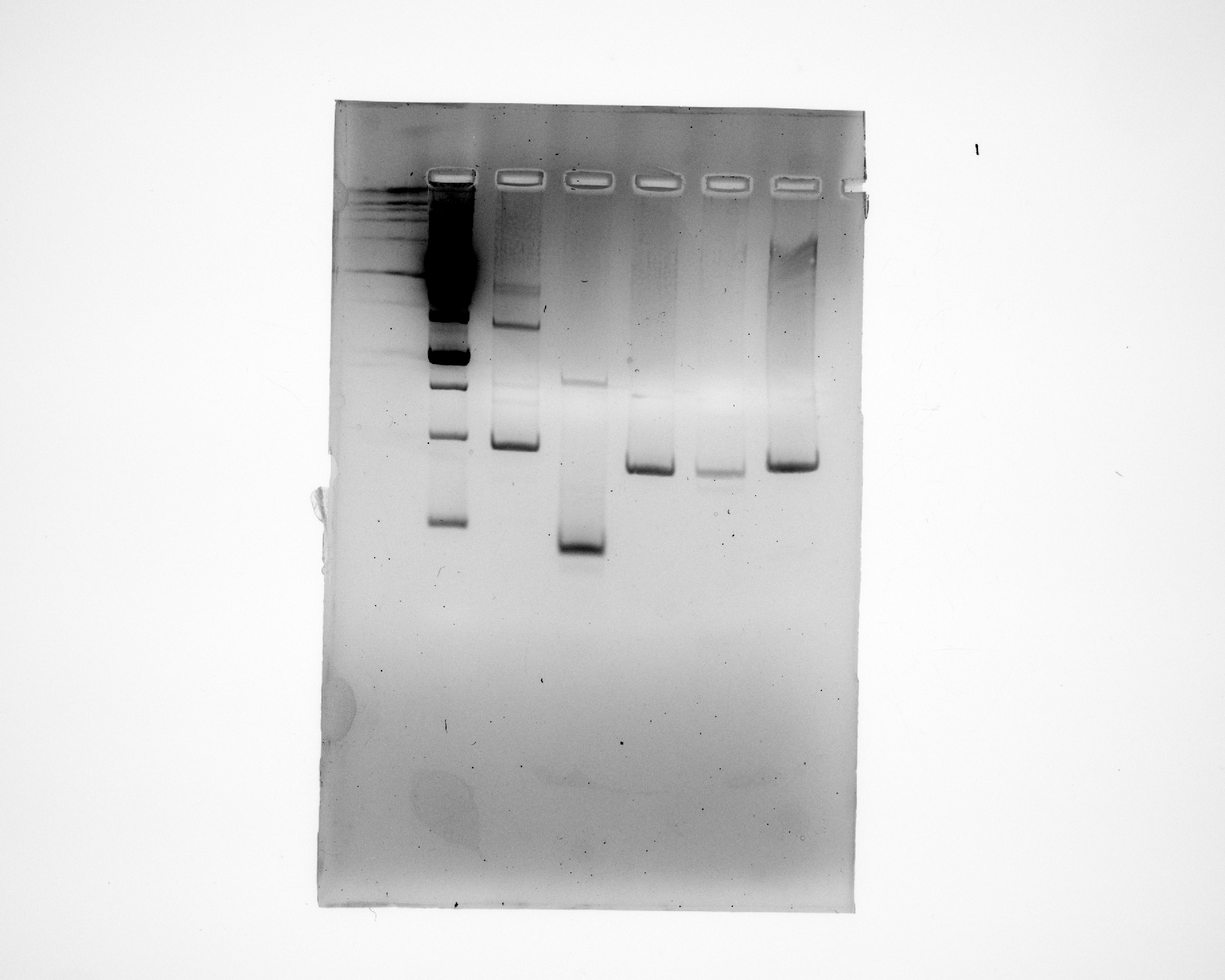


*Candidate 1 2 3 4 5*

500 -

Base pairs

250 -

485

217

427

421

456

**b**

**Potential off-target site 1:**

ACCAGGTCGGAGCTGTGCGAGAGTAGGCGGCCCGCCGCGGAGGCGCGCGCAGGGGTCGGGGGGGGAAACGGGCCCTGGCCCCCGGCTGGAGTAGGGGACGGGGTGCGGGACCCCACCCCACGCCCCGAAAACCAGGCTGCTGCAGTCCCCGGGCTCCACGGACAACTGCCCTGAAAGTCGTTAACACCGAGCGGTGGTTAGTGTGGCGTCGATCGCTCGCAGGTGATCCACGGAGTGCAAGGAGCTTTTGTTTGCACACTAGTGGGCGTGATGGCAGGCCGAGCCTTCTC**CCA**GCCTGGGTTTGGCGGAGGCAGATGACTGAAGGCTGGTCTGGGCGGCCGAGTGGCTATGGAGAGGGACGTCTTTTTCAGGAAGAAGACAGCAGGTGCTGAATTTGGAAACGGTGCTTACTGGCAACGTTAACTGGGATAGGTGCTATTTAGAACCGGCCACGTTACAA

**Potential off-target site 2:**

GGGGCGTCATTGAAGGAGGTGGGTTCCCACTCAGCTACCTGGCCCAGAACCCTCAGCAGGCATTTCCTATCCCCCACAGACCTACTTGTCCTCCTCAGCCAGCCCTGGC**TGG**GGTGCTGCCCTGGAGCTCCCAGTGGAATTCTGCAGGAAGAACACCCTTTCTCTATTTAGTGTCCTTGACCAGAGGGAAAGTGGATCCTCTCGGCTGCCCAGCCCCAAATTTTCCTTGGCTCCAGCGAGGGTGCACTAATTGCAGGCTACTCCCTCCGTCTCGCTGACTGGACCATGGACAGACAATAACCAACCTGACTACGGTGATA

**Potential off-target site 3:**

AAAGAACAGAGGTGGCGCTGGCCCGGTCATCAGGTGTGTGAGGAGGACGCTGGGTCAAGTGGGGGGCTTGGTCTCTAAAATTCAGAACATCTGGCATTTCAAACGTAGGAAAGCTCAGCAAGTCAGCAGACCTGCCGAACACCAAAGCTCCCTGACGAAAAGCTCAGCTTCCAGGTTTGCGGAAATTTGGGAGAGGAACCGAGTTCTTACTGGAATGTGGCCTATCGCTGGCTGACAGATCTGACATGGAATGTCTCCAAATGGCAGTGTCTCCCTCTCTGC**CCT**CCCTGGGAGTGCGCGGAGGAGGAGCAGCCACCGTCCGTGGAGGTGGGCGGGCCTCAGGCAGCCATCTTCGCCAGCTGCTGCTCCAACTTGGAAATGCGCTCGTCTTGACTATAAA

**Potential off-target site 4:**

TTAAGCGGGCCTCGCTGGCGTTCAGGCACCCGGGACCTTAGGCGTCCGCCCGCCGCCGCCTGGCTCTTCCGCCCGCCTTGCTCCCGGCGTGCACCGCGGCGCGGCCATGTTGCCGTAGTGTTGTTTTCTTCCTGCGGAGGCGAGGGTCCGCTGCGGACCGCGACGCGCGGGCCTCGCCTCTGCTCGCTCGCGGGCGCCGGCCCTCGCCGCTCCCCCGCCTCCCCGGCCCGCACCAGAGCCGTCGGCCTGCCAGGAGCTCGGGGGACGGCTGAAGGCCGGGCCCGGGGACCACCATGGCGG**CCG**CCCTGGGCGCAGGCGGAGGAGCAGGCGCTGGAGGTACGTGGGCCGCGGCGGGGGCCGTAGACCGGGCACCCTTTATGTAGGGCCTTCGTGCAAG

**Potential off-target site 5:**

CTCCGGAACTTCGGTTCATCCTCCCAGGGAGGAGAGACGACTGCAGGACTCAGGTGTGGCGCCTTGAATCCGAGCATCATCCGTCAGAGCCTCCTAAGTAGCTCATAGGCCTAATGTGTGAACATTGATTTCTGGGGTGACAGGGAAGCCAGAATGATTTACATCCAATTTCCCATCGATGATATACCATTGATCTCCGAGAGCACTGAAATATTTACTAGGGACAGCAATTCTCGGCCAGGATCTGTCAGCTGGAGGATGAGGCATGCTGGGAAGGATGCGGGATAAGAATAAGATCTCTGGGAATAAGGTCTGAGCTATCCCGGGCCATAGGAGCTGGGAAACCATACGCGGCA**CCA**GGCTGGGTGTGGGGAGGAGAGTGGGACTCTGAGGATTTCCTCTTGATCTCAATAAATGGTGCCCAGGTCAAAAATATCCTCTCAATCTTAAGAAATTGCGTGCAAAATCTTCGTGTTTTTTATCGCCCCCCCTACGCTGGCAACCATCCTGACTCCACGCTGCTGGCAGATTTCCATATGCTCCGCCCCCCTGACGAGCATCCCAATTCTCCCATCCTCAAATCATAGGTGTCGTCACCCGACCTGACTATGAAGATACCCGGTGTTTCCTCCTGTATCCTCCCTCCTGCACTCTCCTCTTCCTAATCCTCAACTTACCGTGATACCCTGTCGGCCTTCTCTCCCTTTCCGTAATACTATGCCCACTATTCTCCATATCT

**Supplementary Fig. S1.** Assessment of off-target cleavage in the genome. Once LH2 KO cells (KO-1) grew to acceptable size, cells were lysed to isolate genomic DNA for PCR amplification and Sanger sequencing. (a) the detection of PCR products amplified by specific primers that contain five potential off-target sites. (b) the sequencing results of PCR products by Sanger sequencing. The potential off-target sites are underlined.


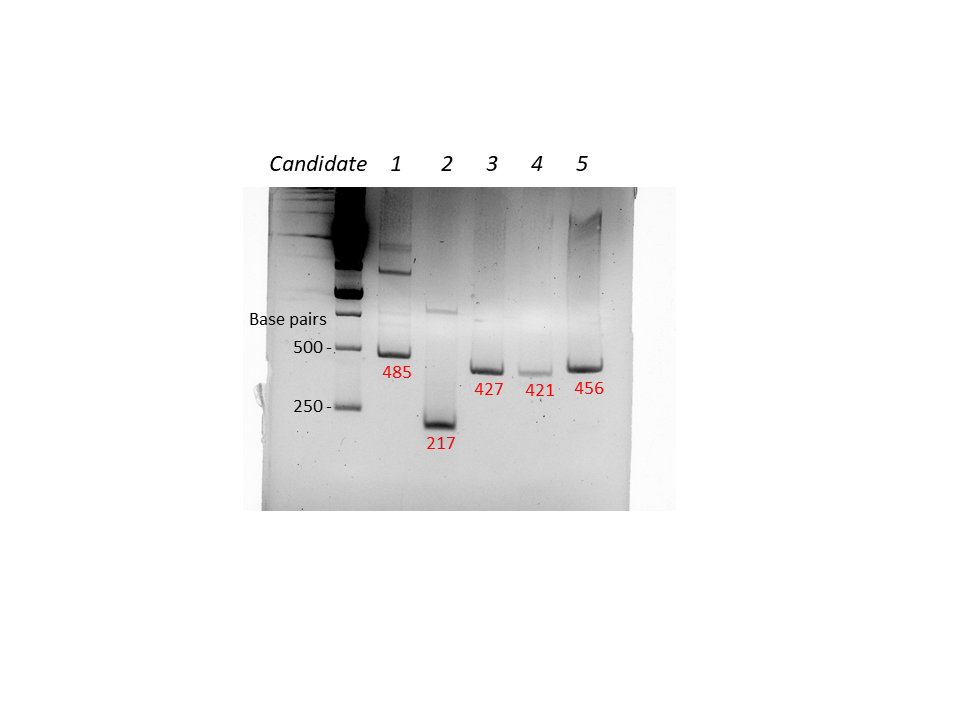


**Supplementary Fig. S2.** Uncropped gel image of PCR products for Sanger sequencing.

**
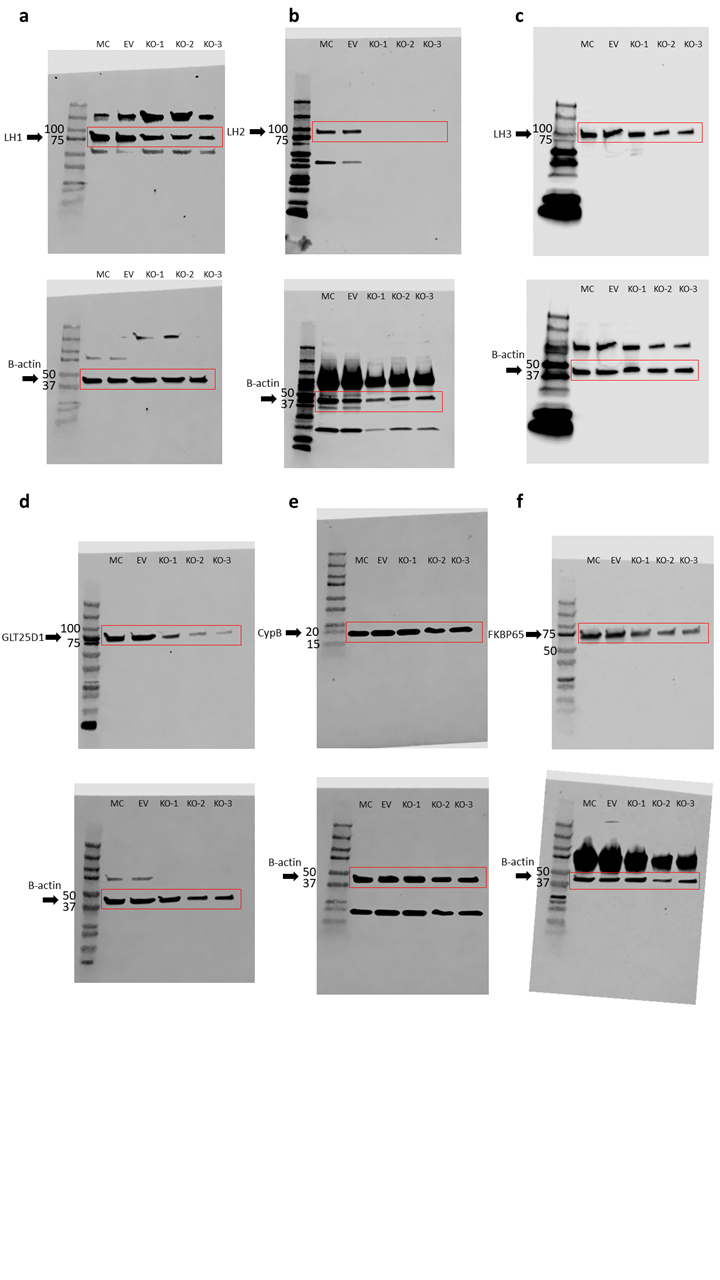
**

**
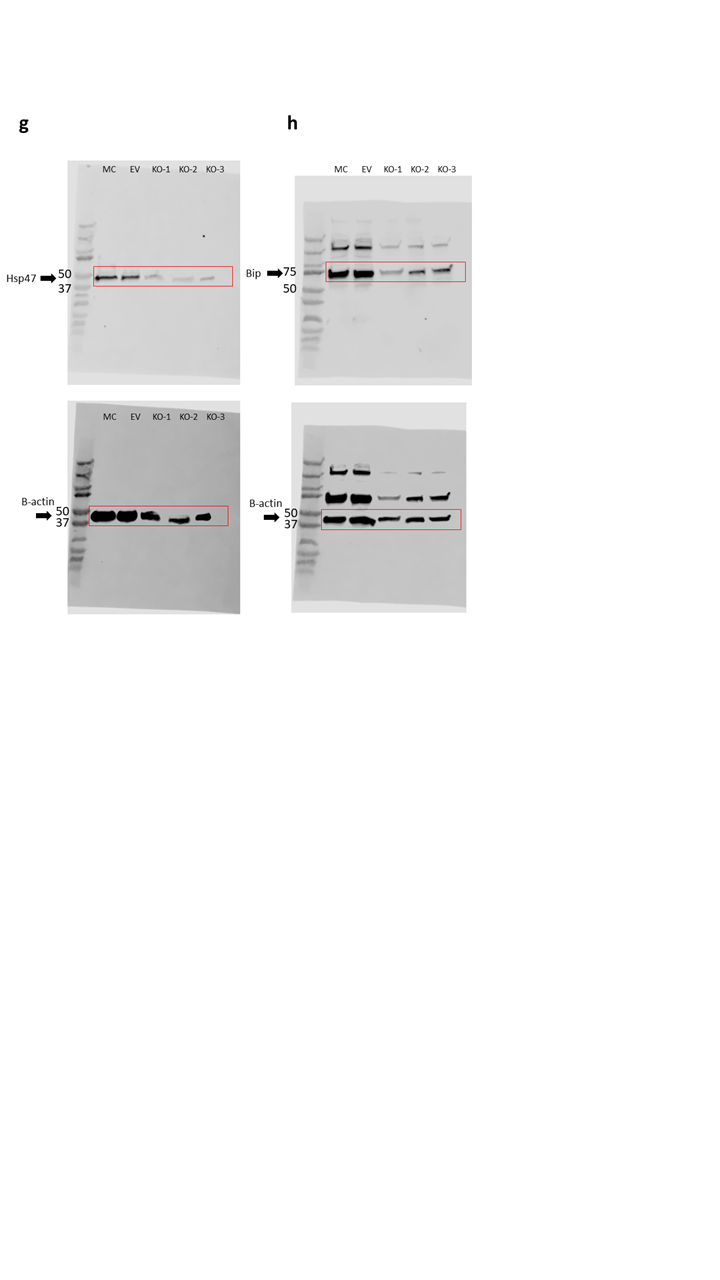
**

**Supplementary Fig. S3.** Uncropped immunoblot images. (a) LH1, (b) LH2, (c) LH3, (d) GLT25D1, (e) CypB, (f) FKBP65, (g) Hsp47, (h) Bip. LH, lysyl hydroxylase; GLT25D1, glycosyltransferase 25 domain containing 1; CypB, cyclophilin B; FKBP65, FK506-binding protein 65; Hsp47, heat shock protein 47; Bip, immunoglobulin heavy-chain-binding protein; MC, MC3T3-E1; EV, empty vector; KO, knock-out.

Mouse Plod2a tccttggctgctcttaagcccgcgccgcgggcgcctccggttccgtcgtccgctccttgc 60

Mouse Plod2b tccttggctgctcttaagcccgcgccgcgggcgcctccggttccgtcgtccgctccttgc 60

Consensus ************************************************************

Amino acid

Mouse Plod2a gctGCCTGGtccgccgaagtccgctcaatcctctccgtgctcttcggtacccacgcagtc 120

Mouse Plod2b gctGCCTGGtccgccgaagtccgctcaatcctctccgtgctcttcggtacccacgcagtc 120

Consensus ************************************************************

Amino acid

Mouse Plod2a ctcccagtccgccctccgctccagccccgggctcacgtctggtcctgcgctcttcggggt 180

Mouse Plod2b ctcccagtccgccctccgctccagccccgggctcacgtctggtcctgcgctcttcggggt 180

Consensus ************************************************************

Amino acid

Mouse Plod2a ctcagcgtctcgcgagaagtcctcgccacaggccttcggctcccgggttcaggggcgg**at** 240

Mouse Plod2b ctcagcgtctcgcgagaagtcctcgccacaggccttcggctcccgggttcaggggcgg**at** 240

Consensus ************************************************************

Amino acid

Mouse Plod2a **g**ggggaccgcggagcgaggccggggcggctgatgcccatgctcgccctgctctcctgggc 300

Mouse Plod2b **g**ggggaccgcggagcgaggccggggcggctgatgcccatgctcgccctgctctcctgggc 300

Consensus ************************************************************

Amino acid M G D R G A R P G R L M P M L A L L S W

**gRNA 1**

**gRNA 2**

Mouse Plod2a gg**ccg**gcctgggcgtggcggaggagacgcccgggcgcatccctgc**agg**tgagctctgctt 360

Mouse Plod2b gg**ccg**gcctgggcgtggcggaggagacgcccgggcgcatccctgc**agg**tgagctctgctt 360

Consensus ************************************************************

Amino acid A A G L G V A E E T P G R I P A D K L L

**Supplementary Fig. S4.** Position of gRNAs used for targeting the mouse *Plod2* gene. The first 360 bp of mouse *Plod2a/2b* genes and their amino acid sequences were aligned. A pair of gRNAs, which target both *Plod2a* and *Plod2b,* were shown as gRNA 1 and gRNA 2, respectively. The “consensus” line indicates conservation of sequence identity. “*” indicates a fully conserved residue. The start codon (atg) was shown in bold. Plod, procollagen-lysine, 2-oxyglutarate, 5-dioxygenase.
